# A mobile laboratory for ancient DNA analysis

**DOI:** 10.1371/journal.pone.0230496

**Published:** 2020-03-18

**Authors:** José Utge, Noémie Sévêque, Anne-Sophie Lartigot-Campin, Agnès Testu, Anne-Marie Moigne, Régis Vézian, Frédéric Maksud, Robert Begouën, Christine Verna, Sylvain Soriano, Jean-Marc Elalouf

**Affiliations:** 1 Unité Eco-anthropologie, Muséum National d’Histoire Naturelle, CNRS, Université Paris Diderot, Paris, France; 2 Université de Lille, Ministère de la Culture et de la Communication, CNRS UMR 8164 Histoire, Archéologie et Littérature des Mondes Anciens, Villeneuve d'Ascq, France; 3 Unité Histoire Naturelle de l’Homme Préhistorique (HNHP), Muséum National d’Histoire Naturelle, CNRS, Université de Perpignan Via Domitia, Centre Européen de Recherches Préhistoriques de Tautavel, Tautavel, France; 4 École d’ingénieurs de Purpan, Institut National Polytechnique de Toulouse, Toulouse, France; 5 Service Régional de l’Archéologie, Toulouse, France; 6 Association Louis Bégouën, Laboratoire de Préhistoire de Pujol, Montesquieu-Avantès, France; 7 Département Homme et Environnement, Unité Histoire Naturelle de l’Homme Préhistorique (HNHP), Muséum National d’Histoire Naturelle, CNRS UMR 7194, Paris, France; 8 CNRS UMR 7041, Maison de l’Archéologie et de l’Ethnologie, Nanterre, France; 9 Université Paris-Saclay, CEA, CNRS, Institute for the Integrative Biology of the Cell (I2BC), Gif-sur-Yvette, France; University of Florence, ITALY

## Abstract

Mobile devices for on-field DNA analysis have been used for medical diagnostics at the point-of-care, forensic investigations and environmental surveys, but still have to be validated for ancient DNA studies. We report here on a mobile laboratory that we setup using commercially available devices, including a compact real-time PCR machine, and describe procedures to perform DNA extraction and analysis from a variety of archeological samples within 4 hours. The process is carried out on 50 mg samples that are identified at the species level using custom TaqMan real-time PCR assays for mitochondrial DNA fragments. We evaluated the potential of this approach in museums lacking facilities for DNA studies by analyzing samples from the Enlène (MIS 2 layer) and the Portel-Ouest cave (MIS 3 deposits), and also performed experiments during an excavation campaign at the Roc-en-Pail (MIS 5) open-air site. Enlène *Bovinae* bone samples only yielded DNA for the extinct steppe bison (*Bison priscus*), whereas Portel-Ouest cave coprolites contained cave hyena (*Crocuta crocuta spelaea*) DNA together, for some of them, with DNA for the European bison sister species/subspecies (*Bison schoetensacki*/Bb1-X), thus highlighting the cave hyena diet. Roc-en-Pail *Bovinae* bone and tooth samples also contained DNA for the *Bison schoetensacki*/Bb1-X clade, and *Cervidae* bone samples only yielded reindeer (*Rangifer tarandus*) DNA. Subsequent DNA sequencing analyses confirmed that correct species identification had been achieved using our TaqMan assays, hence validating these assays for future studies. We conclude that our approach enables the rapid genetic characterization of tens of millennia-old archeological samples and is expected to be useful for the on-site screening of museums and freshly excavated samples for DNA content. Because our mobile laboratory is made up of commercially available instruments, this approach is easily accessible to other investigators.

## Introduction

Over the last twelve years, the field of ancient DNA has experienced deep changes that come both from technological advances and the attention paid to a variety of DNA sources. On the one hand, next generation DNA sequencing methods enabled deciphering complete animal and human genomes [1–3], including the genome of a human lineage not known from the hominin fossil record [4]. On the other hand, the survey of archeological specimens revealed additional and sometimes unexpected sources of ancient DNA such as the avian distal feather components [5] and eggshell [6], dental calculus [7], mollusk shell [8], sediments [9], with especially high yield from hair shafts [10], coprolites [11], and the petrous bone [12]. As a complementary approach, new experimental procedures for DNA extraction [13–15] or sampling [16,17] have been described.

Despite these advances, days to weeks are required from the time a sample has been excavated to the delivery of DNA data. This contrasts with the increasing use of physics methods to perform on-field analysis of archeological material, which enable for example to characterize the pigments of rock art paintings [18–20].

For studies on extant DNA, great attention is paid to on-field analysis to perform medical diagnostics at the point-of-care, for forensic sciences, food testing, environmental monitoring, and detection of biothreat agents (see [21,22] for review). Progress accomplished thanks to such studies for rapid DNA analysis, together with the increasing need to avoid the dissemination of museum collections and the observation that freshly excavated fossils are best for ancient DNA analysis [23,24] call for the evaluation of the on-field approach for ancient DNA studies.

**Fig 1:**
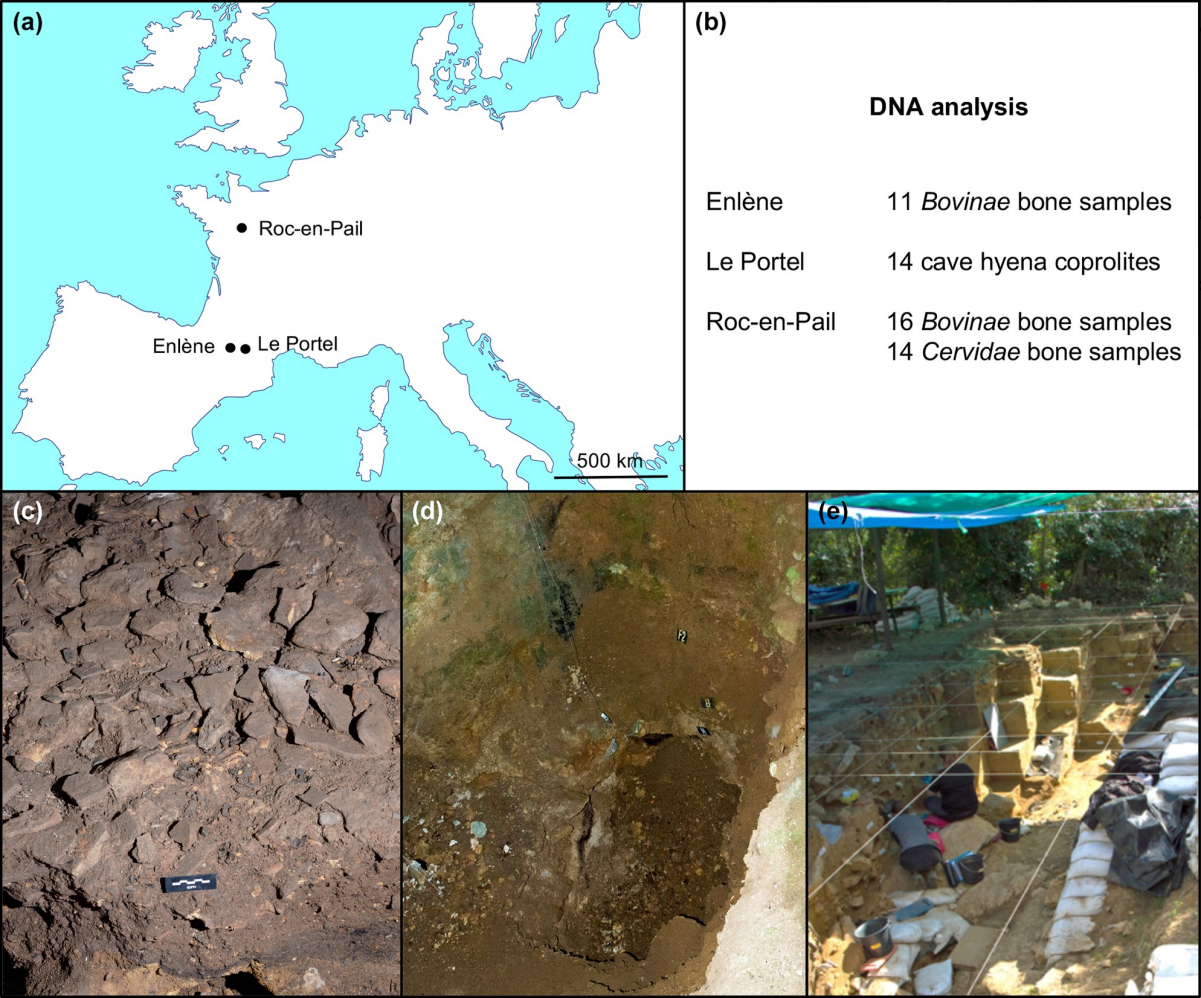
Information about archeological sites. **(a)** Location of sites. **(b)** Samples studied in each site for DNA content. **(c)** Enlène cave. Paving stones in the Salle-du-Fond. **(d)** Portel-Ouest cave. The 5-m thick archeological deposit. **(e)** Roc-en-Pail open-air site during the 2018 excavation campaign.

Because ancient DNA is most often present in trace amounts in archeological samples, contamination by modern or previously amplified DNA is a matter of concern. With this limitation in mind, we attempted to obtain the proof-of-concept for an ancient DNA mobile laboratory by focusing on DNA from extinct species or species no longer present in the locations where our studies were performed. Also, we used the method of real-time PCR because it avoids the post-PCR processing of amplified DNA. We report here on the use of a mobile laboratory consisting of commercially available devices for DNA extraction and amplification, and of TaqMan probes [25] that we designed for real-time PCR analysis of the mitochondrial DNA of six animal species. We conducted studies on samples from three Paleolithic sites: the Enlène cave, the Portel-Ouest cave, and the Roc-en-Pail open-air site (Fig 1).

Enlène (450 m above sea level (asl)), together with the Tuc d’Audoubert and the Trois-Frères caves, is part of the Volp cavern system [26], a property of the Bégouën family located near Montesquieu-Avantès (Ariège, France). By contrast to the Tuc d’Audoubert and the Trois-Frères caves, which are famous for Magdalenian rock art, Enlène contains almost no such art [27,28]. Enlène is nevertheless a major site which provided clues to Magdalenian daily life. Interest for archeological remains in Enlène began in 1860 with excavations performed by canon Jean-Jasques Pouech, was strongly reinforced in 1911 when Jacques Bégouën found a carved propulsion device, and furthermore in 1914 when Jacques and brothers Louis and Max discovered the Trois-Frères cave and its subterranean connection with Enlène [28]. Excavations carried out in the 1920’s and 1930’s by Louis Bégouën [29], then from 1970 to 1990 by Robert Bégouën, Jean Clottes and coworkers revealed 30,000 years of human occupation beginning with the Gravettian period [30]. Notably, these studies yielded abundant Magdalenian tools and portable art pieces. They also provided evidence for the sedentary settlement of magdalenian people in the deep cave sector (the *Salle-du-Fond*) which displays paving stones, bowl-shaped fire structures, and remains of hunted animal species. A palynological study carried out on this level revealed the presence of on open landscape, dominated by *Poaceae* and *Asteraceae*, with steppe species such as *Artemisia*, *Helianthemum*, *Armeria*, and *Rubiaceae*, growing in a cold and dry climate [31]. In the present study, we performed the DNA analysis of *Bovinae* bone remains excavated from the 18,000-year-old (Marine Isotope Stage 2 (MIS2)) Magdalenian layer of the Enlène *Salle-du-Fond*. This was done in the laboratory of the Bégouën Museum in Montesquieu-Avantès.

The cave of Le Portel (490 m asl) is also located in Ariège, near the village of Loubens, about 30 km away from Enlène. The site, a property of the Vézian family, consists of two caves which were connected to each other until a rockfall sealed the communication during MIS 5. The largest cave (eastern entrance) displays Magdalenian and Gravettian rock art pictures that were discovered in 1908 [32]. The Portel-Ouest cave (western entrance), discovered some ten years later, contains a rich, more than 5-m thick archeological sequence. Excavations carried out from 1949 to 1987 in the Portel-Ouest cave by Joseph and Jean Vézian disclosed 21 layers that yielded Neandertal bones and teeth [33], as well as lithic tools and animal remains spread over a time period of about 100,000 years, from MIS 5 to MIS 2 [34,35]. In this study, we analyzed cave hyena coprolites from layers D and B1 of the Portel-Ouest cave, dating back to MIS 3. Occupation by the cave hyena in these levels is well documented by the presence of numerous skeletal remains, coprolites and toothmarks on large mammal remains [36,37]. Layer D yielded Mousterian tools [38] and micro and macrofaunal remains that are consistent with the warming and dampening phase [39,40] of a cold climate, associated with the presence of a semi-open landscape [41]. Layer B1 yielded Upper Paleolithic tools. Faunal remains in this layer indicate a shift from a cold steppe to a partially forested environment [39,40], and the pollen record from north Pyrenean sites for this time period points to the dominance of steppe taxa with a progressive increase of forested taxa with the altitude [41]. Layers D and B1 correspond to the Middle to Upper Paleolithic transition. The archeological remains of Le Portel are stored in the Centre Européen de Recherches Préhistoriques (CERP, Tautavel, France) were we performed the DNA studies.

Roc-en-Pail (Chalonnes-sur-Loire, Maine-et-Loire; 30 m asl) is an open-air site from Western-Central France that is shared between several owners. It is located on the left bank of the Layon River. The site was discovered during the winter of 1870–1871 on the fringes of a quarry supplying lime kilns. Fossil fauna, mainly reindeer, and flint tools were unearthed at the foot of the small Devonian limestone hill exploited by the quarry [42]. The site was excavated by Dr. Gruet from 1943 to 1956 and shortly reopened in 1969. Gruet’s fieldwork was very briefly described in several preliminary publications, and a synthetic description of the stratigraphy was later published [43]. The site was additionally described in studies devoted to sedimentology [44] and the relative chronology is only known from pollen studies [45]. In 2014, new excavations were launched with the aim to provide a comprehensive stratigraphy and establish the chronology. With almost 5-m thick deposits and numerous archeological layers, Roc-en-Pail is the longest sequence described so far for the Middle Paleolithic in Central Western France. Lithic industries are associated with faunal and macrofaunal remains in all layers [46]. Seventeen OSL samples are under processing so the preliminary chronology relies only on geomorphology, fauna and industry record. The bulk of the samples analyzed for DNA content come from stratigraphic unit 401, a sandy loam from the first phase of slope deposits potentially associated with intra-MIS 5 climate degradation, either the MIS 5d or 5b. Mixed fauna recovered in this unit is consistent with the mosaic environments described in deposits from the Early Weichselian in Northern France [47]. It is associated with a Middle Paleolithic industry characterized by recurrent Levallois debitage. The DNA studies of *Bovinae* and *Cervidae* remains were performed in a house next to the site during the 2018 excavation campaign.

## Materials and methods

### Archeological specimens

#### Enlène cave

We studied 11 archeological samples from the Enlène cave. The samples, registered under numbers Enlène 6170 to 6180, are stored in a permanent repository in the Laboratoire de Préhistoire de Pujol, 09200 Montesquieu-Avantès, France. The samples are accessible by others in this private repository upon request to Robert Bégouën at the address indicated above. The permit (reference: MV/FM/MB/2015/20302) obtained for all aspects of the study was delivered by the Service Régional de l’Archéologie (32 rue de la Dalbade, BP811, 31080 Toulouse cedex 6 France), and approved by the collection owner (Robert Bégouën).

#### Portel-Ouest cave

We studied 14 archeological samples from the Portel-Ouest cave. The samples, registered under numbers Portel T1 to T14, are stored in a permanent repository in the Museum of Prehistory reserves, Établissement Publique de Cooperation Culturelle-Centre Européen de Recherches Préhistoriques (EPCC-CERP), 1 avenue Léon-Jean Grégory, 66720 Tautavel, France. The samples are accessible by others upon request at the address indicated above. The permit (reference: 20170328) obtained for all aspects of the study was delivered under the control of the Service Régional de l’Archéologie Occitanie (5 rue Salle-l’Évêque, 34000 Montpellier, France), the collection owner (Régis Vézian), and the administrator of EPCC-CERP.

#### Roc-en-Pail

We studied 30 archeological samples from the Roc-en-Pail site. The samples are registered under numbers Roc-en-Pail 4, 10, 11, 12, 18, 29, 32, 33, 37, 44, 49, 54, 75, 118, 181, 182, 196, 206, 231, 260, 297, 318, 321, 337, 398, 409, 410, 464,528, 529. They are temporarily stored in the research laboratory of the archeological excavation permit holder (Sylvain Soriano, MSH Mondes, UMR7041, 21 allée de l’université, 92000 Nanterre, France). After a period which may not exceed five years after the excavation, the archaeological remains, including the specimens described in this paper, will be deposited and accessible by others in a public repository of the Pays-de-la-Loire region. The location of the permanent repository will be available upon request to the Service Régional de l’Archéologie Pays-de-la-Loire (1 rue Stanislas Baudry, BP 63518, 44035 Nantes Cedex 1, France). The permits (references: 134/2016, 193/2017, 556/2018) for all aspects of the study were delivered by the Prefecture de la region des Pays-de-la-Loire.

### Overview of experimental procedures

The equipment used for DNA studies was carried to museum and archeological sites in the luggage compartment of a mid-size car. In addition to the machines and kits described below that were used for DNA extraction and analysis, small equipment and consumables consisted of tubes, pipettes, pipette filter tips, disposable clothes, surgical blades, Petri dishes, aluminum foil, bench coat protector, biohazard bags, buffers and ultrapure distilled water. The full list of laboratory material is available from the authors. Temperature-sensitive reagents were transported in a cool box and stored at -20°C upon arrival.

Investigators responsible for DNA studies (JME, JU) wore masks, hair nets, disposable lab coats and gloves. Working surfaces were covered with versi-dry protection paper sheets (Nalge Nunc; Rochester, NY, USA) that were changed after each experiment. All material related to the dissection of archeological samples was changed between samples. There was no post-PCR processing of the samples in the museum and archeological sites. Wastes were brought back to the laboratory.

### DNA extraction

For bone samples, we scraped off the outer surface using a single-use surgical blade to delineate a sampling area (S1A and S1B Fig). Then, a clean blade was used to recover bone powder from which DNA extraction was performed. For coprolites, the cortex was removed to allow recovery of material from the coprolite core (S1C and S1D Fig). The samples recovered from coprolites ranged from a fine powder to small granules. When granules were obtained, the sample was crushed between two aluminum foils. For bone and coprolites samples, the powder was transferred into a 2-ml Eppendorf tube (Eppendorf, Hamburg, Germany) until filling the bottom of the tube (S1E Fig). This amount of material corresponds to 30–50 mg. Five hundred μl of DNA extraction buffer (0.25 M EDTA, 10 mM Tris-EDTA (pH 8), 0.2% N-lauryl-sarcosyl, 200 μg/ml proteinase K (Thermo Fisher Scientific (Walthman, MA, USA)) were added to each experimental or mock sample, and the tubes were incubated 2–16 h at 42°C under constant agitation (1,200 rpm) in an Eppendorf thermomixer. The tubes were then centrifuged (5,000 g; 5 min) in an Eppendorf microcentrifuge, and the supernatant was transferred into an Amicon ultra-0.5 ml 30 kDa centrifugal filter unit (Millipore; Burlington, MA, USA). The filter unit was centrifuged (14,000 g; 7 min), rinsed 4 times by adding 500 μl of ultrapure DNase/ RNase-free distilled water (Thermo Fisher Scientific) and centrifugation (14,000 g; 7 min), and the DNA extract was recovered as a ~ 50-μl sample volume by centrifugation (1,000 g; 1 min) of the filter unit in the reverse position. The extract was further purified using Qiagen minelute PCR purification kit (Qiagen; Venlo, Netherlands) according to manufacturer’s instructions, and eluted in 50 μl of 10 mM Tris, pH 8.

### Real-time PCR analysis

Real-time PCR analysis was carried out using TaqMan MGB probes obtained from Thermo Fisher Scientific. Primers and probes for the TaqMan assays (Table 1) were designed with the help of Primer Express software 3.0.1 (Thermo Fisher Scientific) or the custom assay design tool available on the Thermo Fisher Scientific web site using reference sequences for the mitochondrial genomes of *Bison priscus* (NC_027233), *Bison schoetensacki* (NC_033873), *Rangifer tarandus* (NC_007703), *Cervus elaphus* (NC_007704), and *Crocuta crocuta spelaea* (NC_020670). Real-time PCR was carried out in a 20-μl reaction volume containing 10 μl of 2X TaqMan fast advanced master mix (Thermo Fisher Scientific), 900 nM of forward and reverse primers, 250 nM of TaqMan probe, and water (PCR blank), mock or DNA extracts. The TaqMan fast advanced master mix contains dUTP (instead of dTTP) and uracil-N-glycosylase to prevent from carryover contamination from one experiment to another. For each sample, 3 to 4 serial dilutions corresponding to 2 to 0.03 μl of the DNA extract were usually analyzed. Amplification was performed in a Mic PCR cycler (Bio Molecular Systems; Upper Coomera, Australia) and included the following steps: 50°C, 2 min; 95°C, 20 s; then 40 PCR cycles (95°C, 3 s; 60°C, 30 s). Data were analyzed using Mic PCR software set with default parameters to determine the cycle threshold (*C*_T_), *i*.*e*. the number of PCR cycles required for the fluorescent signal to exceed the background level. Only samples for which a *C*_T_ value below or equal to 37 cycles were considered positive for DNA content.

**Table 1:** Primers and probes used in this study.

Application	Species	Oligonucleotide sequence	Target, size (bp)
TaqMan	*Bison priscus*	F, CCCCAGCAAATCCACTCAATACA	CYTB, 81
		R, TTGATCGTAAAATTGCGTATGCAAATAAG	
		Probe, CCTCCCCACATCAAAC	
TaqMan	*Bison schoetensacki*	F, CTACTAGTACTATTCGCACCCGA	CYTB, 75
		R, AGGCGTGTTAAGTGGATTTGCT	
		Probe, CCTCCTCGGAGACCCAG	
TaqMan	*Bos primigenius*	F, CCAGCCAATCCACTCAACACA	CYTB, 79
		R, TTGATCGTAAGATTGCGTATGCAAA	
		Probe, CCCCTCACATCAAACC	
TaqMan	*Rangifer tarandus*	F, TCACATCTGTCGAGACGTCAATT	CYTB, 61
		R, TGCTCCGTTGGCATGTATGTA	
		Probe, TGGCTGAATCATCCG	
TaqMan	*Cervus elaphus*	F, CATGTAGGGCGAGGCCTGTA	CYTB, 66
		R, AACTACTCCGATGTTTCACGTCTCT	
		Probe, ACGGATCATATACTTTTC	
TaqMan	*Crocuta crocuta*	F, TGGCGAGACATTATCCGAGAA	COX3, 68
		R, CGCAGCCCCTTTTGTACAGT	
		Probe, CACATTCCAGGGACACC	
PCR-sequencing	*Bison priscus*	F, CCAAACCCTTAAACTATCCCTC	ND5, 74
		R, CGAATAGTGCTACTGGGACA	
PCR-sequencing	*Bison schoetensacki*	F, CTACTAGTACTATTCGCACCCGA	CYTB, 75
		R, AGGCGTGTTAAGTGGATTTGCT	
PCR-sequencing	*Rangifer tarandus*	F, ATCCGATACAATAACAGCAT	CYTB, 65
		R, TTCAGCCATAATTGACGTCT	

Oligonucleotides sequences are displayed in the 5’-3’ orientation. F, forward PCR primer; R, reverse PCR primer; Probe, TaqMan minor groove binder (MGB) probe labeled with 6-carboxyfluorescein (FAM). The last column indicates the region of the mitochondrial genome amplified with each primer set and the size of the amplicon.

### Kinetic analysis of DNA recovery

These experiments were performed in the Paleogenomic and Genetic analysis platform of the Musée de l’Homme. This platform has a clean room facility for pre-PCR steps carried out on ancient DNA, and modern DNA laboratories for PCR and post-PCR steps.

To set up a rapid DNA extraction procedure, we used the methods described above to analyze two samples previously published: a steppe bison rib [48], and a cave hyena coprolite [11]. Briefly, 300 mg of bone or coprolite powder was recovered using a single-use surgical blade, divided in two 150 mg samples that were transferred into 2-ml Eppendorf tube, and 1.5 ml of DNA extraction buffer was added. The samples were incubated under constant agitation in an Eppendorf Thermomixer, and aliquots of 500 μl were retrieved after a 1-h, 2-h, or 16-h (sample 1) or a 1-h, 3-h, or 16-h (sample 2) incubation time. DNA was then extracted using Amicon and Qiagen columns and eluted in 50 μl of 10 mM Tris, pH 8. Sizing of the DNA recovered in extracts was performed by electrophoresis using the LabChip GX touch nucleic acid analyzer (PerkinElmer; Waltham, MA, USA). TaqMan assays for steppe bison, cave hyena, and red deer DNA were performed using the Mic PCR cycler.

### DNA sequencing

To confirm that correct species identification was achieved using TaqMan assays, a subset of DNA extracts were further analyzed by DNA sequencing. In order to avoid dissemination of amplified DNA in museum and archeological sites lacking an appropriate laboratory, these studies were performed in the Paleogenomic and Genetic analysis platform of the Musée de l’Homme.

Aliquots (0.3 to 0.9 μl) of the DNA extracts were PCR-amplified in a 50-μl reaction volume containing 300 nM of forward and reverse primers (Table 1), 200 μM dNTP, 2.5 mM MgCl_2_, 5 μl of GeneAmp AmpliTaq Gold DNA polymerase buffer II, and 2.5 U of AmpliTaq Gold DNA polymerase (Thermo Fisher Scientific). To disclose the DNA damages expected for authentic ancient DNA, uracil-N-glycosylase was not present in the PCR mix used for these experiments. PCR procedures consisted in an enzyme activation step (95°C, 10 min), followed by a single round of 45 PCR cycles (95°C, 15 s; 53°C, 20 s; 70°C, 1 min) performed in a Veriti thermal cycler (Thermo Fisher Scientific). The full reaction volume was loaded onto an 8% polyacrylamide gel stained with Sybr Green I (Thermo Fisher Scientific). PCR blanks and mock extracts always failed to yield any amplification products. PCR amplicons were eluted from the gel, recovered in 10 mM Tris (pH 8), and ligated to Illumina (San Diego, CA, USA) adapters. Libraries of DNA fragments were generated using the Illumina TruSeq Nano DNA LT sample kit following the manufacturer’s recommendations, except that 5 PCR cycles were performed after the ligation step. Illumina sequencing of pooled amplified libraries was performed on the MiSeq platform by Fasteris (Geneva, Switzerland). The sequence reads were quality filtered, the adapter sequences were removed using Fastx-Toolkit 0.0.13 (http://hannonlab.cshl.edu/fastx_toolkit/download.html) and Cutadapt (version 2.7) [49] with a quality score of 20 and above, and minimum read length of 50 nucleotides. Sequence reads were aligned using CodonCode Aligner v.8.0 (http://www.codoncode.com/aligner/), and DNA sequence logos were generated using the seqLogo R package [50]. The DNA reads of this study have been deposited at Dryad (doi:10.5061/dryad.8gtht76jz). For each sample, the consensus sequence derived from DNA reads was used for species identification by BlastN alignment to the sequences recorded in the GenBank nr/nt database.

## Results

### Mobile DNA laboratory

**Fig 2:**
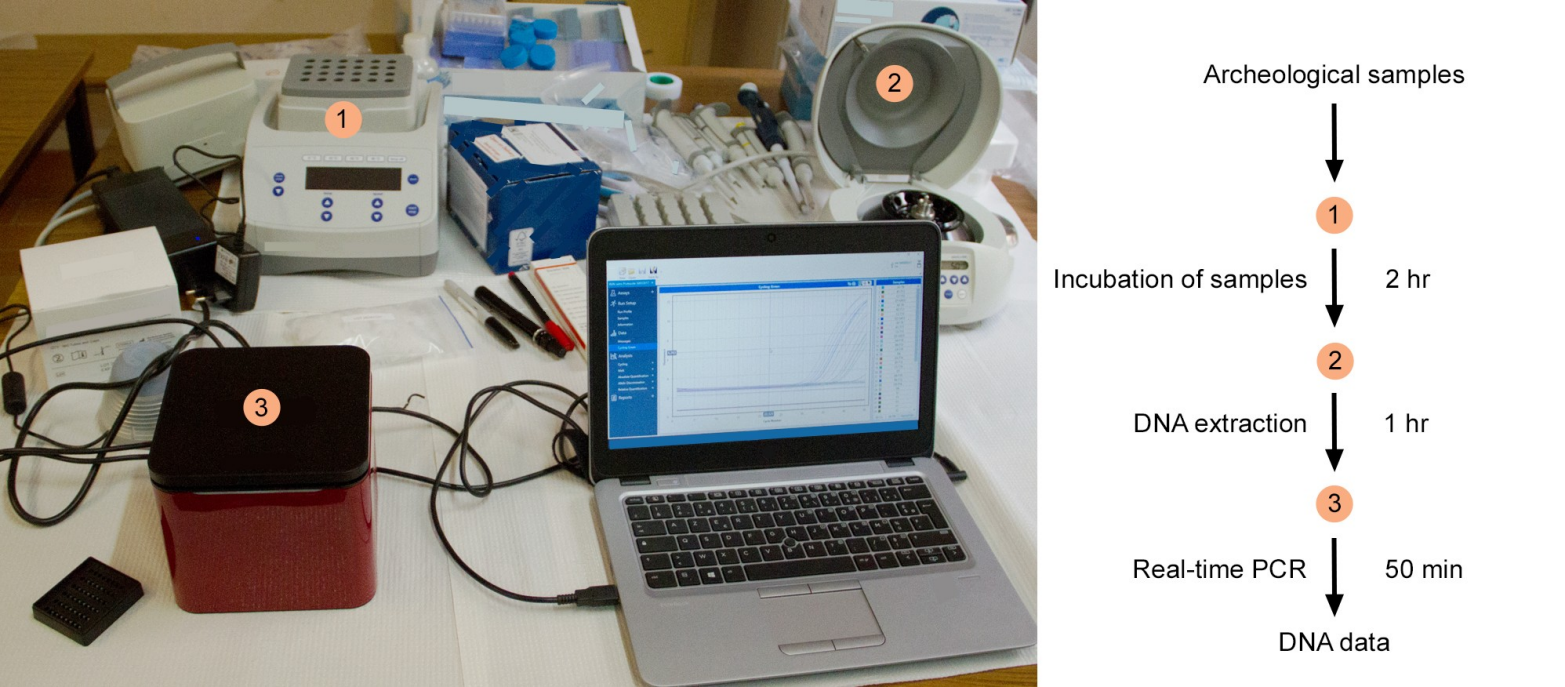
Overview of the device and experimental workflow. The left panel shows the heater-mixer used for sample incubation (1), the mini-centrifuge used for DNA extraction (2), and the real-time PCR instrument (3). The right panel summarizes the overall experimental procedure.

Fig 2 shows the mobile DNA laboratory and key steps for DNA studies. The entire process, from archeological samples to DNA data, can be performed within 4 hours: a 2-h incubation time (see below), a 1-h extraction step, and a 50-min real-time PCR run. Our device allows to perform DNA extraction from 12 samples (including mock extracts) at a time. Then, up to 4 replicates of all 12 samples can be analyzed simultaneously with the appropriate TaqMan probe in the 48 reaction tubes of the Mic real-time PCR cycler.

In this study, we analyzed Pleistocene remains of herbivore (*Bovidae*, *Cervidae*) and carnivore (*Hyaenidae*) species. Late Pleistocene Eurasian specimens of the cave hyena display some particular features, including different body size and proportions as compared to the most closely related extant species, the African spotted hyena (*Crocuta crocuta*) [51,52]. Consequently, the species/subspecies status of the cave hyena is debated and has been alternatively referred to as *Crocuta crocuta*, *Crocuta spelaea*, or *Crocuta crocuta spelaea*. However, genetic studies revealed that the Eurasian cave hyena and the extant spotted African hyena are so closely related that the cave hyena should be considered as a chronospecies rather than a distinct species or even subspecies [11,53]. We therefore refer below to cave hyena remains as *Crocuta crocuta* samples.

### Validation of experimental procedures

**Fig 3:**
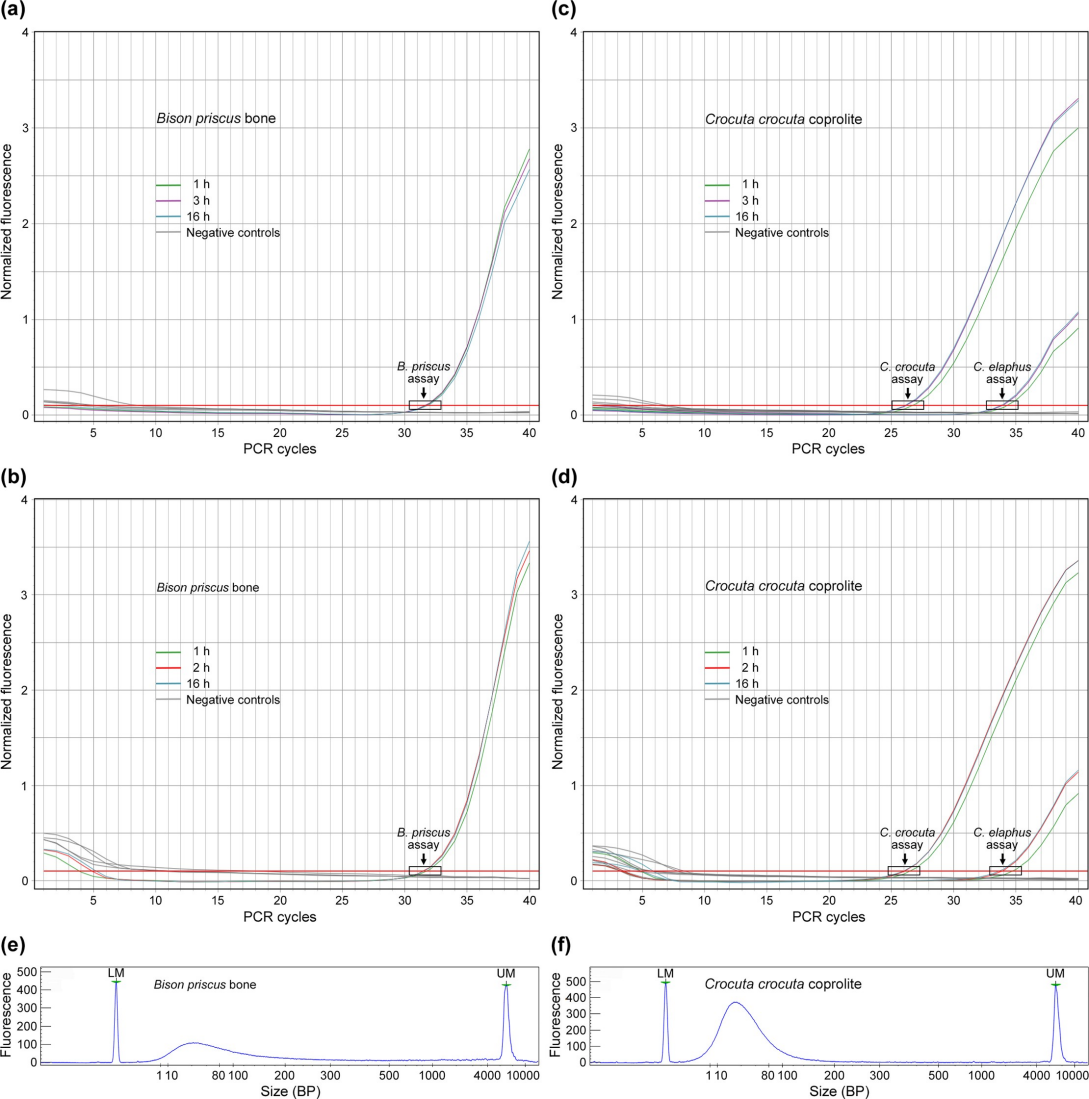
Validation of experimental procedures. **(a, b)** Real-time PCR analysis of a steppe bison (*Bison priscus*) bone sample [48] incubated 1 h, 3 h or 16 h **(a)** and 1 h, 2 h, or 16 h **(b)** before being processed for DNA extraction. The green, purple, blue, and red amplification plots display results obtained using 1 μl of the steppe bison DNA extracts with the *Bison priscus* TaqMan assay. Negative controls: PCR blank, mock and cave hyena DNA extracts (1 μl) analyzed using the *Bison priscus* TaqMan assay. **(c, d)** Real-time PCR analysis of a cave hyena (*Crocuta crocuta*) coprolite [11] incubated 1 h, 3 h or 16 h **(c)** and 1 h, 2 h, or 16 h **(d)** before being processed for DNA extraction. The green, purple, blue, and red amplification plots display results obtained using 1 μl of the coprolite DNA extract with TaqMan assays for *Crocuta crocuta* and *Cervus elaphus* DNA, as indicated. Negative controls: PCR blank, mock and bison bone DNA extracts (1 μl) analyzed using the same TaqMan assays. **(e, f)** Electrophoregrams for the *Bison priscus* bone sample (**e**, 1-μl aliquot) and the *Crocuta crocuta* coprolite (**f**, 0.3-μl aliquot) DNA extracts obtained after a 2-h incubation period. LM: lower marker; UM; upper marker.

To demonstrate the potential of our method to perform ancient DNA studies, we analyzed two samples previously published: a steppe bison rib (SGE2) from which a complete mitochondrial genome sequence for the steppe bison has been deciphered using shotgun DNA sequencing [48]; and a cave hyena coprolite (CC8) from which shotgun DNA sequencing yielded a complete cave hyena and a partial red deer (*Cervus elaphus*) mitochondrial genome sequence [11]. For each sample, we performed two experiments: one experiment aiming to compare the DNA extracts recovered after a 1-h, 3-h, or 16-h incubation period; and one experiment aiming to compare extracts recovered after a 1-h, 2-h, or 16-h incubation period. Fig 3A and 3B show that for the *Bison priscus* bone sample, there were no clear-cut differences between the amplification plots for extracts obtained after a long (2 h or more) or a short (1 h) incubation period. For the *Crocuta crocuta* coprolite, *C*_T_ values for DNA amplification were similar for extracts obtained after a 3- to 16-h (Fig 3C) or a 2- to 16-h (Fig 3D) incubation period, and slightly but systematically lower than *C*_T_ recorded after a 1-h incubation period. The data therefore indicate a much better yield of DNA from the coprolite after a long than a short (1 h) incubation period, and no improvement in yield for incubation times longer than 2 h. Importantly, the TaqMan assays carried out on the coprolite extracts made it possible to detect DNA originating from the defecator (*Crocuta crocuta*) and from its diet (*Cervus elaphus*). We also characterized the DNA extracts by analyzing the size of the native (*i*.*e*. unamplified) DNA fragments recovered using our extraction procedure. As expected for ancient DNA, the bulk of the material recovered corresponded to DNA fragments shorter than 100-bp (Fig 3E and 3F). Overall, the data reported in Fig 3 indicate that our extraction procedure and TaqMan assays are suitable for analyzing ancient DNA, and that a 2-h incubation time is long enough for ancient DNA recovery from bone and coprolite samples. Based on these data, we decided to perform incubation times lasting 2 to 16 h for DNA extraction.

### Analysis of Enlène cave samples

The first floor of the Bégouën museum (Montesquieu-Avantès, Ariège, France) includes a room for archiving archeological and paleontological remains excavated from the Volp caverns, and a laboratory in which no ancient DNA studies have ever been performed and where we installed our mobile laboratory. We analyzed 11 *Bovinae* bone samples originating from the Magdalenian layer of the *Salle-du-Fond*. The samples were studied using TaqMan assays for *Bison priscus*, *Bison schoetensacki*, and *Bos primigenius* mitochondrial DNA.

**Fig 4:**
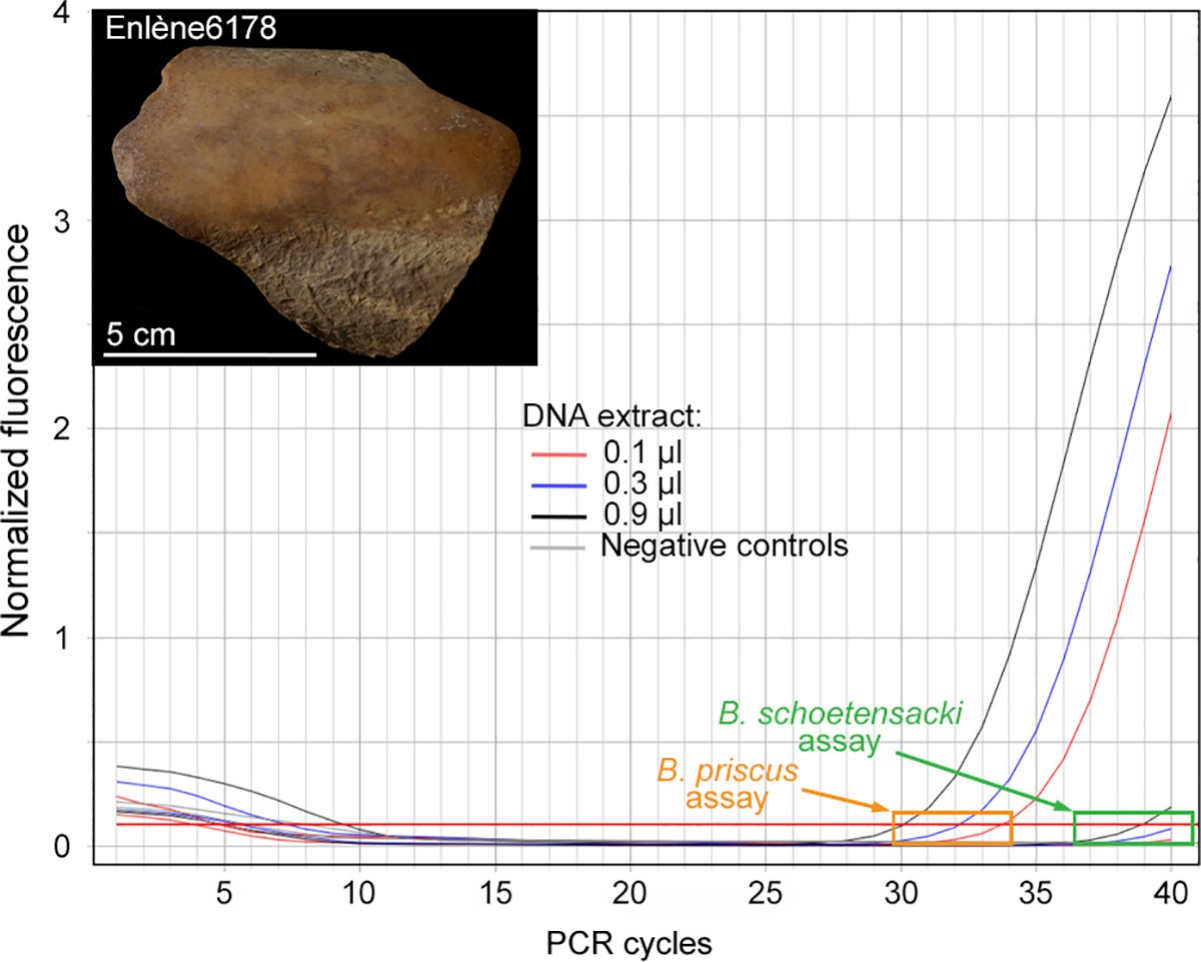
DNA analysis of a bone sample from the Enlène cave. The figure shows real-time PCR data obtained from serial dilutions (from 0.1 to 0.9 μl) of the DNA extract using the *Bison priscus* and the *Bison schoetensacki* TaqMan assays. The distinctive amplification plots obtained with the two assays indicate that the sample corresponds to a *Bison priscus* bone. Negative controls: PCR blank and mock extract.

Fig 4 shows real-time PCR data for the Enlène 6178 specimen. Using the *Bison priscus* probe, amplification was recorded for all three replicate samples, with *C*_T_ values ranging between 30 and 34 cycles, depending on the amount (from 0.9 to 0.1 μl) of DNA extract introduced in the assay. These results indicate robust amplification data for the detection of *Bison priscus* DNA. Fig 4 also shows that, using the same DNA extract, the *Bison schoetensacki* assay yielded either no amplification or a very faint signal, with a *C*_T_ of 39 cycles for the largest amount of extract being used. The large difference between the *C*_T_ values (9 PCR cycles) recorded with the two probes for the same DNA amount indicates efficiencies that differ by more than two orders of magnitude to detect DNA in the extract, thus validating the design of the *Bison priscus* assay. As a whole, for the 11 Enlène cave samples tested, we only obtained evidence for *Bison priscus* DNA, with a total of six positive samples (S2 Fig, S1 Table). For one sample (E6179), two DNA extracts were prepared and studied on separate days, and both of them were positive for *Bison priscus* DNA content.

**Fig 5:**
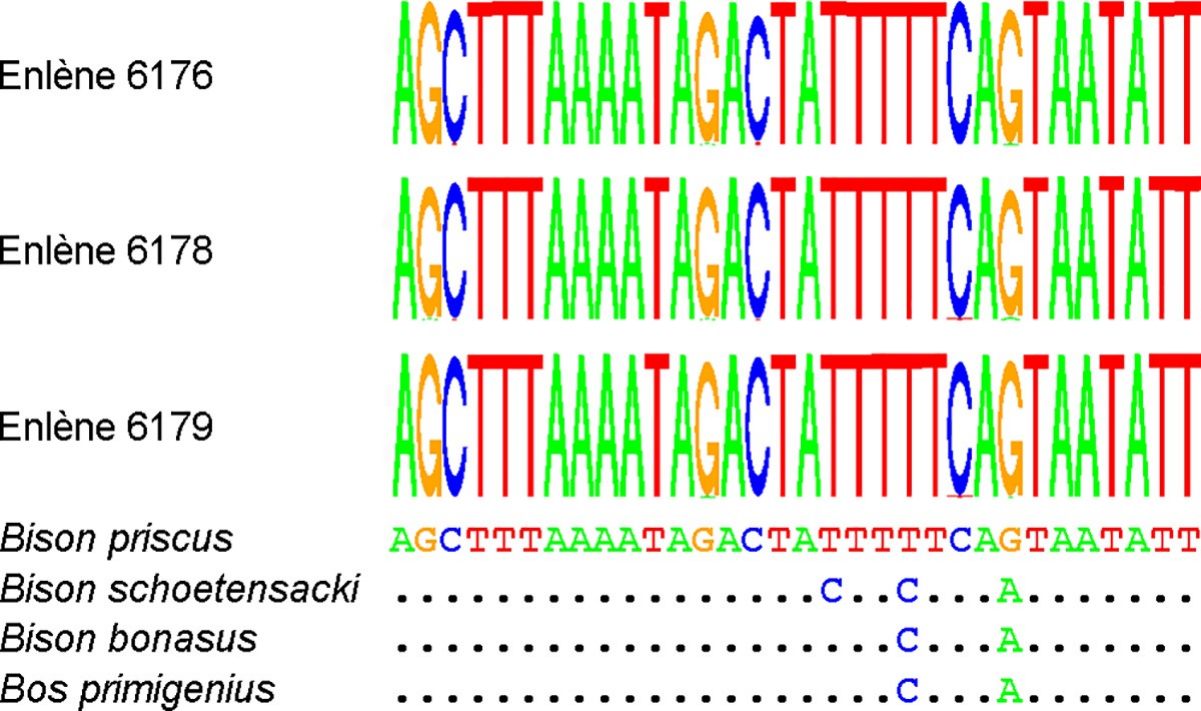
DNA sequence data for Enlène samples. The upper part of the figure displays sequence logos derived from 27,058 (Enlène 6176), 19,361 (Enlène 6178), and 29,097 (Enlène 6179) DNA reads. Only the sequence located between the PCR primers is shown. At each position, the overall height of the stack indicates the sequence conservation, and the height of the letters the relative frequency of the nucleotides; the upper letter corresponds to the predominant nucleotide. The lower part of the figure shows the orthologous reference sequences of the *Bison pricus* (NC_027233), *Bison schoetensacki* (NC_033873), *Bison bonasus* (NC_014044), and *Bos primigenius* (NC_013996) mitochondrial genomes. For each Enlène sample, the consensus sequence is identical to the *Bison priscus* reference sequence. Dots indicate sequence identity.

To confirm that correct species identification was achieved using the TaqMan assay, a subset of Enlène samples were analyzed using high-throughput sequencing of PCR amplicons. The DNA sequencing approach also offers the opportunity to demonstrate that the extracts contain genuine ancient DNA. Indeed, amplicons obtained from ancient DNA display typical patterns of nucleotide misincorporations, that mostly consist in transitions, especially C/G-to-T/A transitions [54]. Fig 5 shows that for each of the Enlène samples analyzed, the consensus sequence perfectly aligned with the *Bison priscus* reference sequence, and that the patterns of nucleotide misincorporations fit with those expected for PCR fragments obtained from ancient DNA. Thus, the sequence data support the conclusions derived from the TaqMan studies.

### Analysis of cave hyena coprolites from the Portel-Ouest cave

We performed the analysis of coprolites from the Portel-Ouest cave in the CERP of Tautavel. CERP, a building contiguous to the Musée de Préhistoire de Tautavel, houses a variety of devices for the analysis of archeological material, but no DNA studies have been performed previously in this laboratory. MIS 3 layers D (end of Middle Paleolithic) and B (Upper Paleolithic) of the Portel-Ouest cave yielded several dozens of *Crocuta crocuta* coprolites, of which 14 (seven from each layer) were selected for our studies.

**Fig 6:**
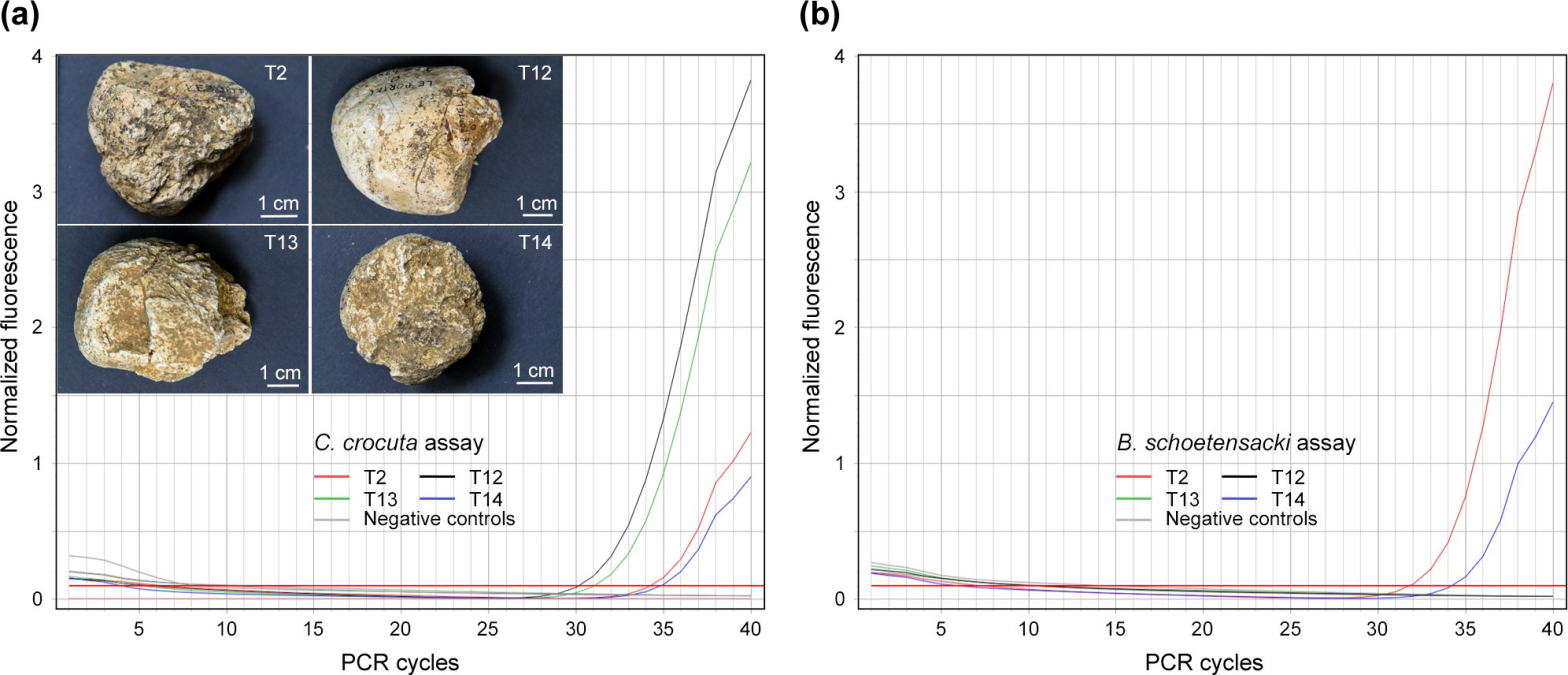
DNA analysis of cave hyena (*Crocuta crocuta*) coprolites from the Portel cave. **(a)** Real-time PCR data obtained with the *Crocuta crocuta* TaqMan assay using 2 μl of each DNA extract. Negative controls correspond to PCR blank and mock DNA extract. **(b)** Real-time PCR data obtained with the *Bison schoetensacki* TaqMan assay using 2 μl of the same DNA extracts. Negative controls: PCR blank and mock extract.

To test for DNA preservation in the cave hyena coprolites, we analyzed the DNA extracts using the *Crocuta crocuta* mitochondrial genome TaqMan assay. As shown in Fig 6A, we observed differential DNA yield from one coprolite to another, with *C*_T_ values ranging between 30.1 (T12 sample, layer B1) and 34.8 (T14, layer B1) PCR cycles. T13 (layer B1) and T2 (layer D) samples returned intermediate *C*_T_ values. Among the 14 coprolites tested, the presence of cave hyena DNA was demonstrated in 8 samples (S3 Fig, S1 Table), including 7 samples from layer B1, and a single sample from layer D. This indicates better DNA preservation in the Upper than in the Middle Paleolithic layer.

**Fig 7:**
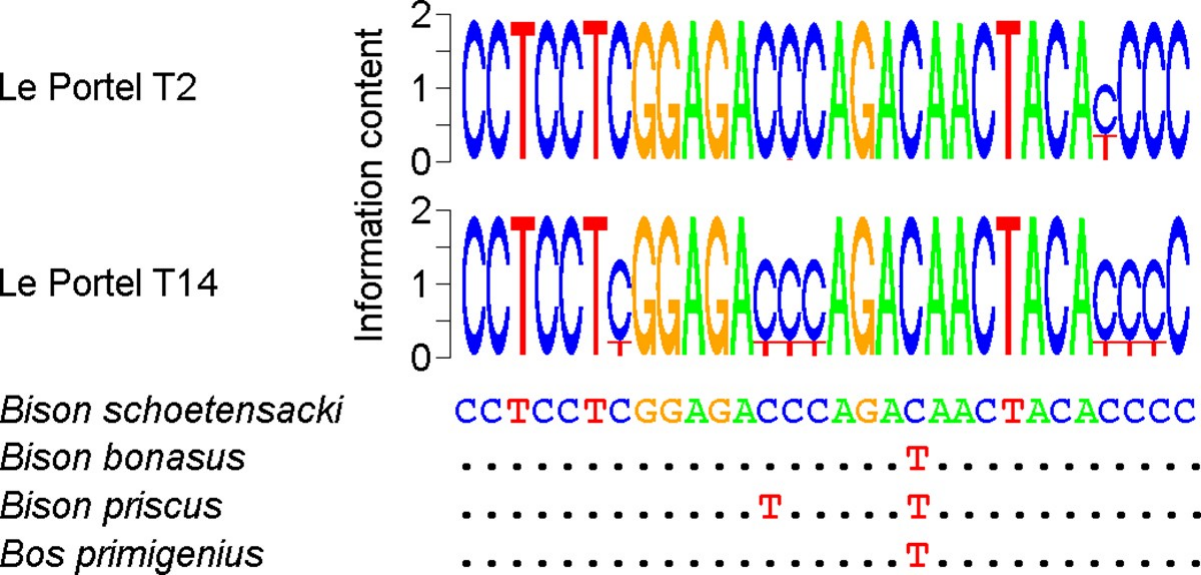
DNA *Bovinae* sequence data for Le Portel samples. The upper part of the figure displays sequence logos derived from 26,130 (Le Portel T2) and 55,101 (Le Portel T14) DNA reads. Only the sequence located between the PCR primers is shown. At each position, the upper letter corresponds to the predominant nucleotide. The lower part of the figure shows the orthologous reference sequences of the *Bison schoetensacki* (NC_033873), *Bison pricus* (NC_027233), *Bison bonasus* (NC_014044), and *Bos primigenius* (NC_013996) mitochondrial genomes. For the each Le Portel sample, the consensus sequence is identical to the *Bison schoetensacki* reference sequence. Dots indicate sequence identity.

To gain some information on the cave hyena diet, we next analyzed all 8 coprolites positive for *Crocuta crocuta* DNA with TaqMan assays for five *Cetartiodactyla* species, including two *Cervidae* and three *Bovinae* species. Whereas no evidence was obtained for the presence of *Cervus elaphus*, *Rangifer tarandus*, *Bison priscus* or *Bos primigenius* in these Portel cave coprolites, amplification of *Bison schoetensacki* DNA was demonstrated in two of them (Fig 6B, S1 Table). Considering the close genetic proximity between *Bison schoetensacki* and *Bison bonasus* [55], additional PCR studies were performed to characterize by DNA sequencing the fragments amplified with the *Bovinae* primers. In the *Bison schoetensacki* mitochondrial genome sequence, the sequence located between the TaqMan primers displays a C residue at a position (nucleotide 15,273 in the reference sequence) where a T residue is recorded in all extant and extinct specimens of the *Bison bonasus* Bb2 [56,57] lineage analyzed so far. The consensus sequences obtained for coprolites DNA amplified using *Bovinae* primers display the distinctive *Bison schoetensaki* C residue (Fig 7). At other positions, abundant C to T substitutions guarantee that amplification was initiated from ancient DNA. The detection of *Bison schoetensaki* DNA in two coprolites, one (T2) recovered from layer D, and one (T14) from layer B1, indicates that this *Bovinae* species was present by the end of the Middle Paleolithic and the beginning of the Upper Paleolithic (*i*.*e*. about 40,000 years ago) in the vicinity of the Portel cave.

### Analysis of Roc-en-Pail samples

The fieldwork carried out at Roc-en-Pail gave us the opportunity to perform ancient DNA studies on freshly excavated samples. Based on paleontological criteria, the bulk of excavated remains are ascribed to *Bovinae* and *Cervidae* specimens. As usual for *Bovinae* remains, species identification was hardly feasible using morphometric data, so most samples were referred to as *Bos*/ *Bison* remains. These samples were systematically analyzed using the three *Bovinae* TaqMan assays. On the other hand, the *Cervidae* remains were expected to correspond to reindeer specimens. They were nevertheless all studied using the *Rangifer tarandus* and *Cervus elaphus* mitochondrial genome TaqMan assays.

**Fig 8:**
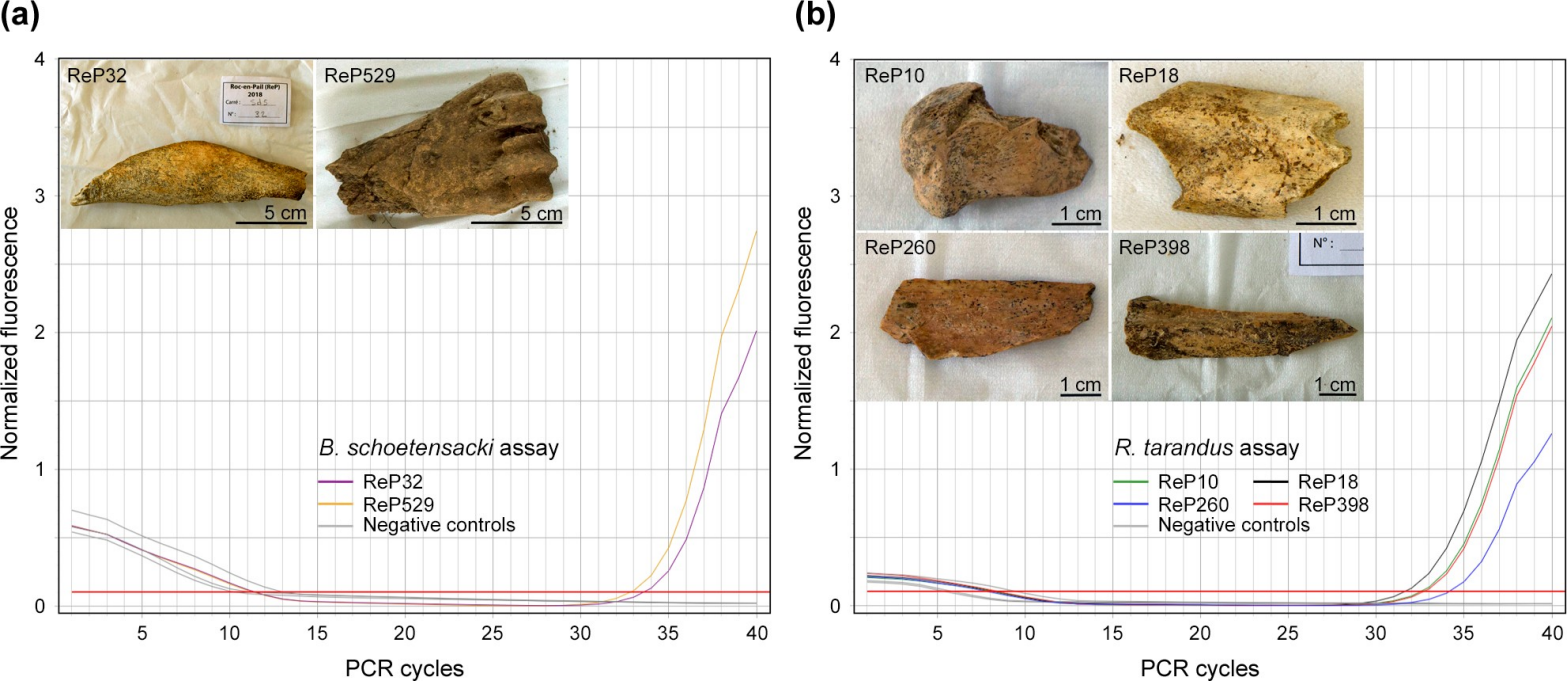
DNA analysis of Roc-en-Pail bone samples during an excavation campaign **(a)** Analysis of Roc-en-Pail 32 and 529 samples using the *Bison schoetensacki* TaqMan assay. **(b)** Analysis of Roc-en-Pail 10, 18, 260, and 398 samples using the *Rangifer tarandus* TaqMan assay. All assays were performed using 0.9 μl of DNA extract. Negative controls: PCR blank and mock extract.

Fig 8A shows results obtained for two *Bovinae* samples using the *Bison schoetensacki* assay. *C*_T_ values with this assay were 32.7 (ReP529) and 33.5 (ReP32) PCR cycles. Of the 16 *Bovinae* samples surveyed, 4 (3 bone fragments and a tooth sample) yielded DNA that could be amplified using the *Bison schoetensacki* assay (S4 Fig), but none of them provided evidence for the presence of *Bison priscus* or *Bos primigenius* DNA (S1 Table). In line with studies performed on Le Portel cave samples (see above), we checked by DNA sequence analysis the correct identification of the DNA fragments for two samples. As shown in S5A Fig, the consensus sequences were identical with the *Bison schoetensacki* reference sequence, and the patterns of nucleotide misincorporations demonstrated the presence of ancient DNA in the extracts.

From a paleontological point of view, Roc-en-Pail bison bone fragments 32, 337, and the deeply worn tooth (ReP 182) do not convey useful information. The fourth Roc-en-Pail sample (ReP 529) in which *Bison schoetensacki* DNA was found corresponds to the distal end of a metatarsal bone. It is not possible from this single bone fragment to obtain a clear-cut identification of the bison species from which it comes from. However, comparison of the metric values of this bone with those of bison specimens from Siréjol, where *Bison schoetensacki* has been recorded [55], indicates that the distal Roc-en-Pail bison metatarsal positions within the Siréjol population (S6 Fig). The large size of this distal metatarsal suggests it belongs to a male individual.

Turning to Roc-en-Pail *Cervidae* remains, Fig 8B shows examples of samples for which successful DNA amplification was achieved using the *Rangifer tarandus* assay. For the whole series of *Cervidae* remains tested for DNA content (14 samples), TaqMan analysis disclosed the presence of *Rangifer tarandus* DNA in 7 bone fragments (S7 Fig, S1 Table), whereas *Cervus elaphus* DNA was never detected. ReP 18 specimen was studied using two DNA extracts obtained on separate days, and both of them indicated the presence of *Rangifer tarandus* DNA. Sequencing analysis confirmed the correct identification of *Rangifer tarandus* DNA using our TaqMan assay (S5B Fig).

## Discussion

This work evaluated the feasibility of using a mobile platform to perform ancient DNA analysis in museums lacking facilities for genetic studies, and as a complementary approach to archeological fieldwork during an excavation campaign. The samples surveyed originate from cave and open-air sites spanning a 60,000-year time period, from MIS 5 (Roc-en-Pail) to MIS 2 (Enlène). Successful real-time DNA analysis was achieved for several animal species using a variety of remains, including coprolite, bone and tooth samples.

Several lines of evidence support the notion that our method provides reliable data, notably that contamination and specificity issues were adequately addressed. First, negative controls (*i*.*e*. PCR blanks and mock extracts) always failed to yield an amplification signal. Second, the species for which DNA amplification was obtained are either extinct or no longer present in the vicinity of the sites where the studies were conducted. Third, when a sample evaluated positive for DNA content was studied through two extracts, the second experiment corroborated the first one. Fourth, DNA sequencing carried out using the TaqMan assay primers or different primers confirmed that correct species identification had been achieved with the real-time PCR assays.

Our mobile platform consists of two devices for DNA extraction (heater-mixer, minicentrifuge) and of a compact real-time PCR machine. In order to make easily accessible the on-field approach, we paid special attention to set-up a platform that only includes commercially available devices. The full set of instruments and pipettes of our mobile platform amounts to 15,000 €, and the cost to perform DNA extraction and real-time PCR analysis (4 replicates) with one TaqMan assay is 15 €. The methods described here allow a single investigator to perform DNA extraction and analysis of 12 samples within 4 hours. The procedures for sample preparation and DNA extraction were designed to limit the number of instruments in the platform and the working time. Thus, instead of a drilling machine we used single-use surgical blades to scrap the bone surface and recover bone powder. This sampling procedure is consistent with the recommendation to retrieve material from the compact bone surface for best endogenous DNA yield [17]. To speed up the DNA extraction process and limit sample handling, we did not perform a bleach wash nor repeated incubation steps [14,15]. Instead, we used a single incubation step procedure and a similar extraction protocol for bone, tooth and coprolite samples. Thus, there is room for improving the on-field procedures for DNA extraction, while keeping in mind that sample handling and working time should be reduced as much as possible. For studying samples from animal species still present in the site environment, bleaching of the archeological material and implementing the platform with a portable clean room is recommended. For human samples, it would not be reasonable to perform on-field ancient DNA studies.

The samples we screened originate from sites that have not been studied previously for ancient DNA. In Enlène, we focused on *Bovinae* remains from the deep cave sector that was inhabited by Magdalenian people 18,000 years ago. We detected *Bison priscus* DNA in several bone samples, which provides a molecular support to confirm the notion that Magdalenian individuals from Enlène hunted the extinct steppe bison. For the Portel-Ouest cave, the analysis of cave hyena coprolites indicated better DNA preservation in the ~ 35,000-year-old (Upper Paleolithic) than in the ~ 40,000-year-old (Middle Paleolithic) layer. Moreover, we were able to detect *Crocuta* as well as *Bison* DNA in some coprolites, indicating that our approach is useful to obtain genetic data on a carnivore and its diet. Interestingly, the bison DNA present in coprolites points to a species different from the one detected in Enlène. Paleontological data argue for the presence of *Bison priscus mediator* remains in the Portel-Ouest deposits, especially in the ~ 45,000-year-old F layer [35]. Thus, comparison of the paleontological and genetic data suggests the presence of several bison species in the cave paleoenvironment, possibly at different time periods (see below). Finally, Roc-en-Pail challenged our method for the analysis of MIS 5 (*i*.*e*. more than 71,000-year-old) samples from an open-air site. While the presence of reindeer remains indicates good agreement between the molecular and archeozoological data, the bison species disclosed at Roc-en-Pail by genetic studies was not fully anticipated and extends the observation made on the Portel cave samples.

Recent studies attempted to elucidate the descent of the European bison or wisent (*Bison bonasus*) by genomic analysis of remains sampled across Eurasia. Their complete mitochondrial genome sequences revealed two clades [55–57]. One clade, referred to as Bb2 by Massilani et al. [56], includes the extant wisent as well as all historical and Holocene specimens and part of the Pleistocene ones. The other clade only includes Pleistocene specimens. The status of this clade is debated. It has been named clade Bb1 or X by the two groups of investigators who consider it as an extinct *Bison bonasus* lineage [56,57]. We proposed that this clade corresponds to the extinct woodland bison (*Bison schoetensacki*) but failed to convince others who rather adhere to the notion that *Bison schoetensacki* became extinct during the Middle Pleistocene [58]. Whatever the exact status of the intriguing *Bison schoetensacki*/Bb1-X clade, in the present studies we recorded it for time periods and paleoenvironments that fit with previous expectations [56,57]. Thus, it was present in the MIS 5 Roc-en-Pail and in the MIS 3 Portel-Ouest layers. Mixed faunal record in these layers suggest the presence of non-continuous deciduous forest as well as a steppe environment that hosted “non-analogue” faunal communities as compared to those of present day [59]. By contrast, in the Enlène Magdalenian habitat (MIS 2 glacial period), we recorded *Bison priscus* specimens.

In conclusion, this work demonstrates the feasibility to perform ancient DNA analysis under a variety of working condition, including nearby an excavation site. The real-time PCR approach we used provides ancient DNA data within a few hours without on-site post-PCR processing of the samples, a prerequisite to avoid the dissemination of amplified DNA in sensitive environments. The robustness of the data was corroborated by subsequent DNA sequencing analysis. With the ongoing progress of methods for DNA analysis, including portable sequencing devices [60], we anticipate great interest in the near future for on-field ancient DNA studies.

## Supporting information

S1 Fig Recovery of bone and coprolite material for DNA extraction. **(a, b)** *Rangifer tarandus* bone sample (ReP49) from Roc-en-Pail. **(a)** Native bone sample; **(b)** the black line delineates the area from which the superficial cortex was scraped off before retrieving bone material from the red-circled zone for DNA extraction. **(c, d)** *Crocuta crocuta* coprolite (T7) from the Portel cave. **(c)** Native coprolite sample; **(d)** a cortical fragment has been removed to recover material from the coprolite core for DNA extraction. **(e)** Bone powder used for DNA extraction. Scale bars: 1 cm.
(JPG)

S2 Fig Enlène cave bone samples successfully analyzed for *Bison priscus* DNA. Scale bars: 5 cm.
(JPG)

S3 Fig Portel cave coprolites successfully analyzed for cave hyena (*Crocuta crocuta*) DNA. Scale bars: 1 cm.
(JPG)

S4 Fig Roc-en-Pail bone samples successfully analyzed for *Bison schoetensacki* DNA. Scale bars: 5 cm.
(JPG)

S5 Fig DNA sequence data for Roc-en-Pail *Bovinae* and *Cervidae* samples. **(a)** *Bovinae* samples. The upper part of the figure displays sequence logos derived from 33,267 (Roc-en-Pail 32) and 30,091 (Roc-en-Pail 529) DNA reads. Only the sequence located between the PCR primers is shown. At each position, the upper letter corresponds to the predominant nucleotide. The lower part of the figure shows the orthologous reference sequences of the *Bison schoetensacki* (NC_033873), *Bison pricus* (NC_027233), *Bison bonasus* (NC_014044), and *Bos primigenius* (NC_013996) mitochondrial genomes. For both Roc-en-Pail samples, the consensus sequence is identical to the *Bison schoetensacki* reference sequence. Dots indicate sequence identity. **(b)** *Cervidae* sample. The upper part of the figure displays sequence logos derived from 23,481 (Roc-en-Pail 18) and 2,229 (Roc-en-Pail 398) DNA reads. The lower part of the figure shows the orthologous reference sequences of the *Rangifer tarandus* (NC_007703), *Cervus elaphus* (NC_007704), *Capreolus capreolus* (NC_020684), *Dama dama* (NC_020700) mitochondrial genomes, and the orthologous sequence of the *Megaloceros giganteus* (AM182645) mitochondrial cytB gene. For both Roc-en-Pail samples, the consensus sequence is identical to the *Rangifer tarandus* reference sequence.
(JPG)

S6 Fig Comparative sizes of metatarsal distal ends of Roc-en-Pail 529 (orange dot) and Siréjol (blue dots) bison specimens.
(JPG)

S7 Fig Roc-en-Pail bone samples successfully analyzed for *Rangifer tarandus* DNA. Scale bars: 1 cm.
(JPG)

S1 Table Summary of samples analyzed and TaqMan assays performed in this study. (DOCX)
