# Supplementary figures and images for: A mobile laboratory for ancient DNA analysis
Source: PLoS One. 2020 Mar 18;15(3):e0230496. doi: 10.1371/journal.pone.0230496 (PMC7080343; doi:10.1371/journal.pone.0230496)

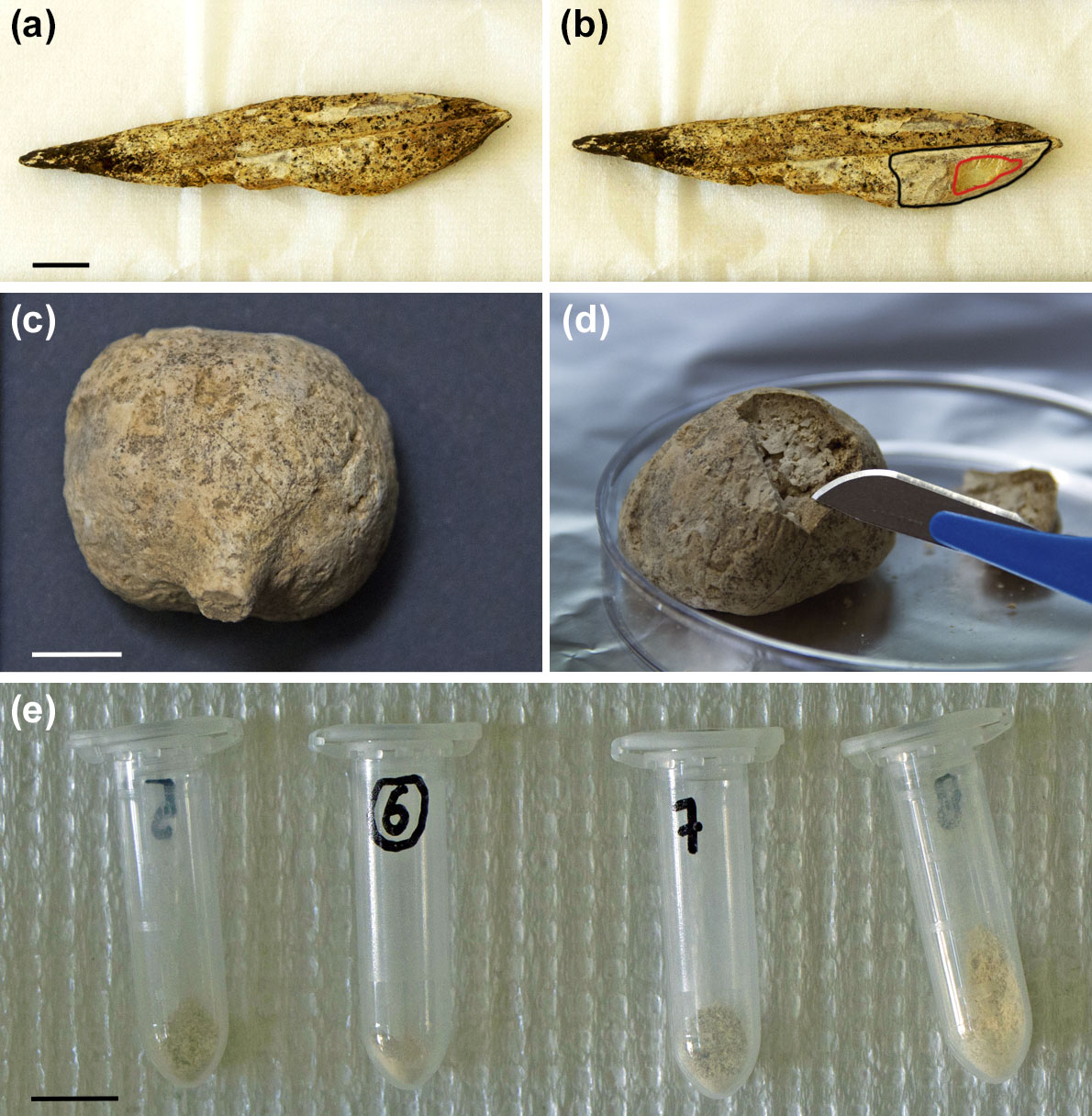

Supplement: S1 Fig — (a, b) Rangifer tarandus bone sample (ReP49) from Roc-en-Pail. (a) Native bone sample; (b) the black line delineates the area from which the superficial cortex was scraped off before retrieving bone material from the red-circled zone for DNA extraction. (c, d) Crocuta crocuta coprolite (T7) from the Portel cave. (c) Native coprolite sample; (d) a cortical fragment has been removed to recover material from the coprolite core for DNA extraction. (e) Bone powder used for DNA extraction. Scale bars: 1 cm. (JPG) [file pone.0230496.s001.jpg]

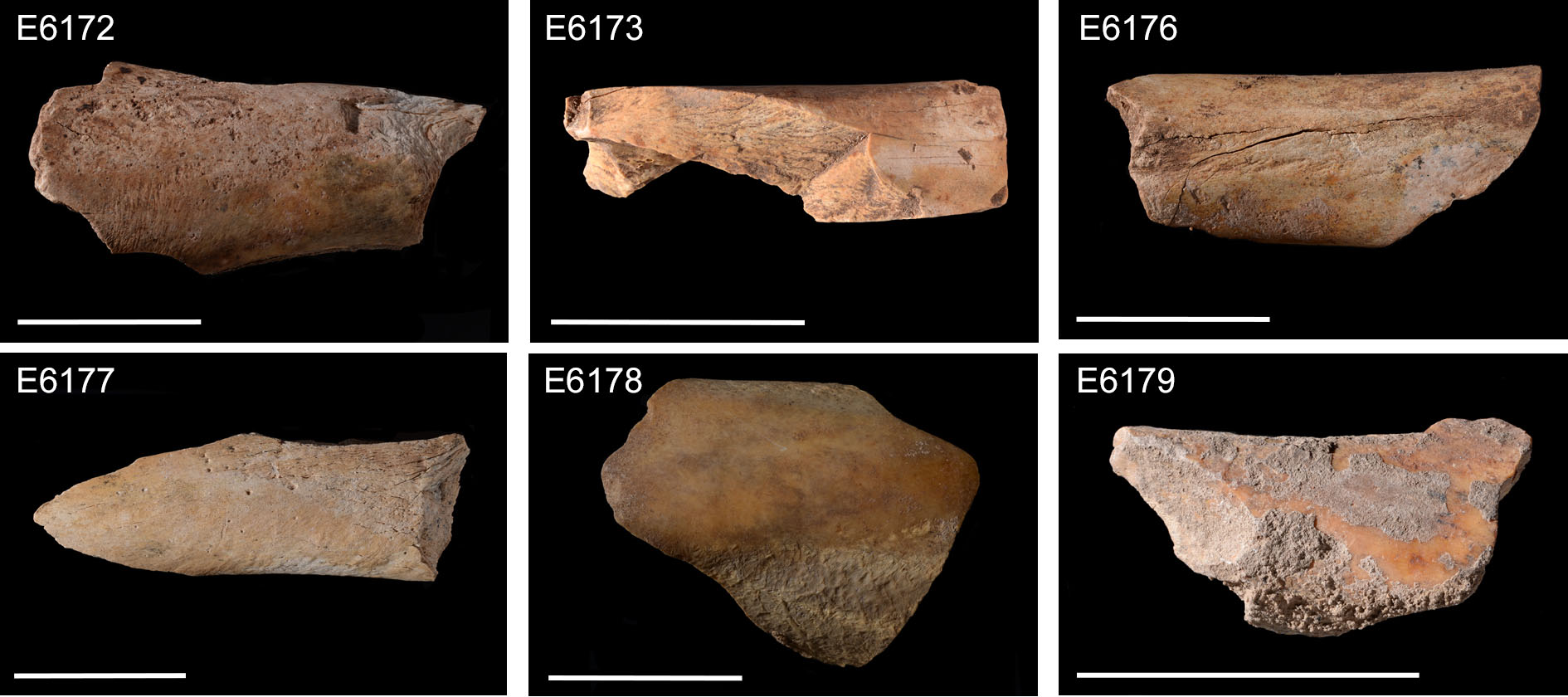

Supplement: S2 Fig — Scale bars: 5 cm. (JPG) [file pone.0230496.s002.jpg]

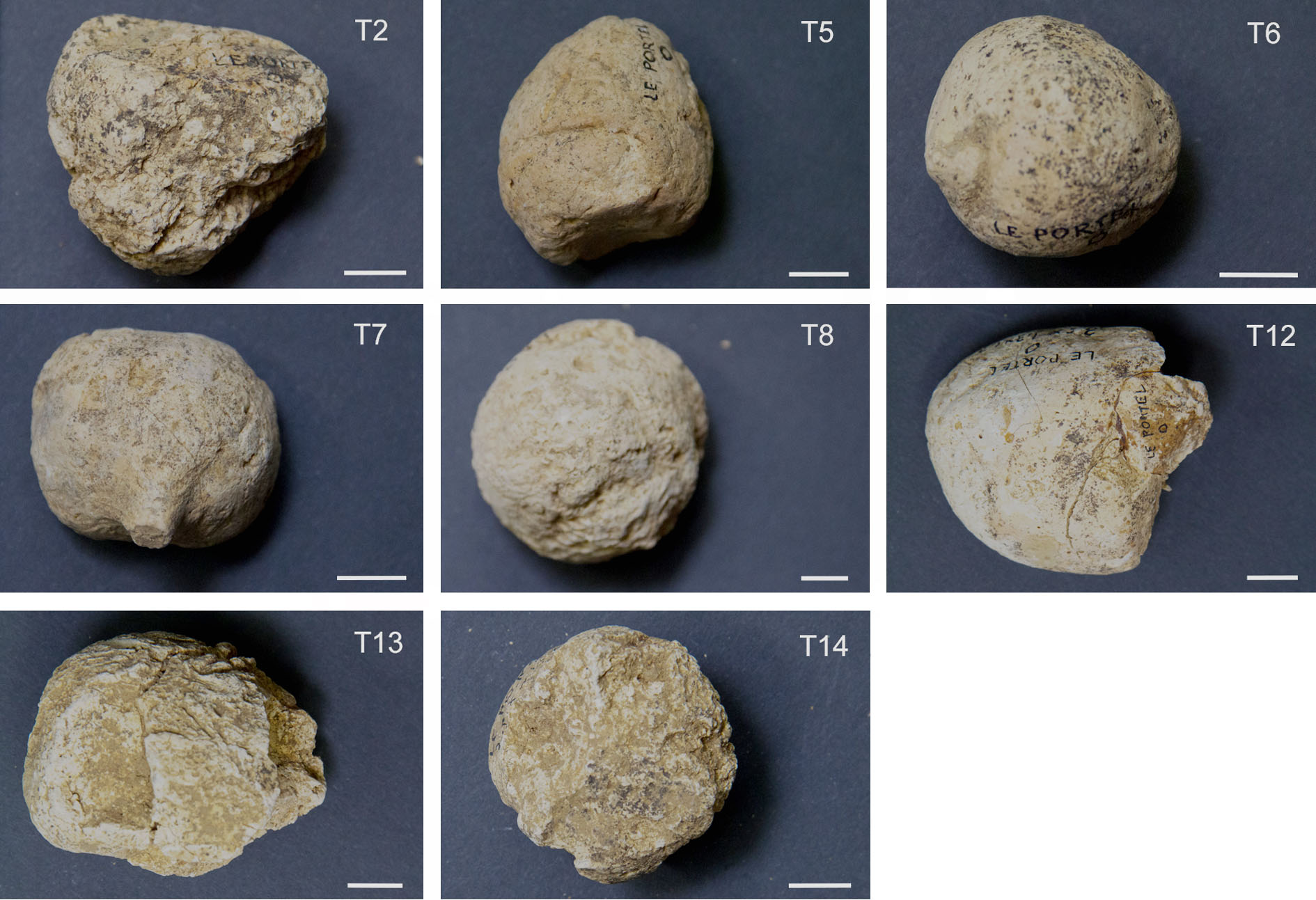

Supplement: S3 Fig — Scale bars: 1 cm. (JPG) [file pone.0230496.s003.jpg]

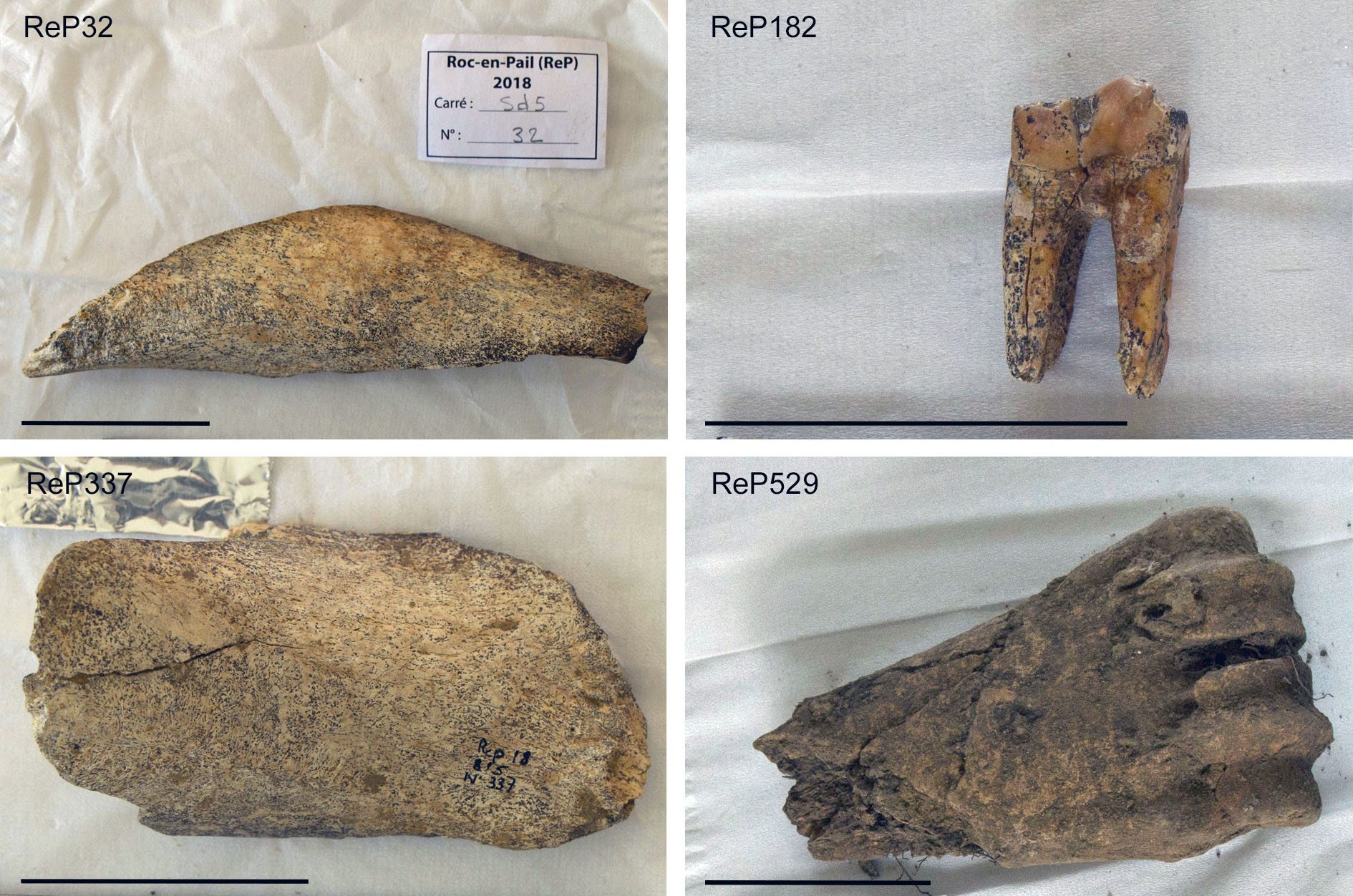

Supplement: S4 Fig — Scale bars: 5 cm. (JPG) [file pone.0230496.s004.jpg]

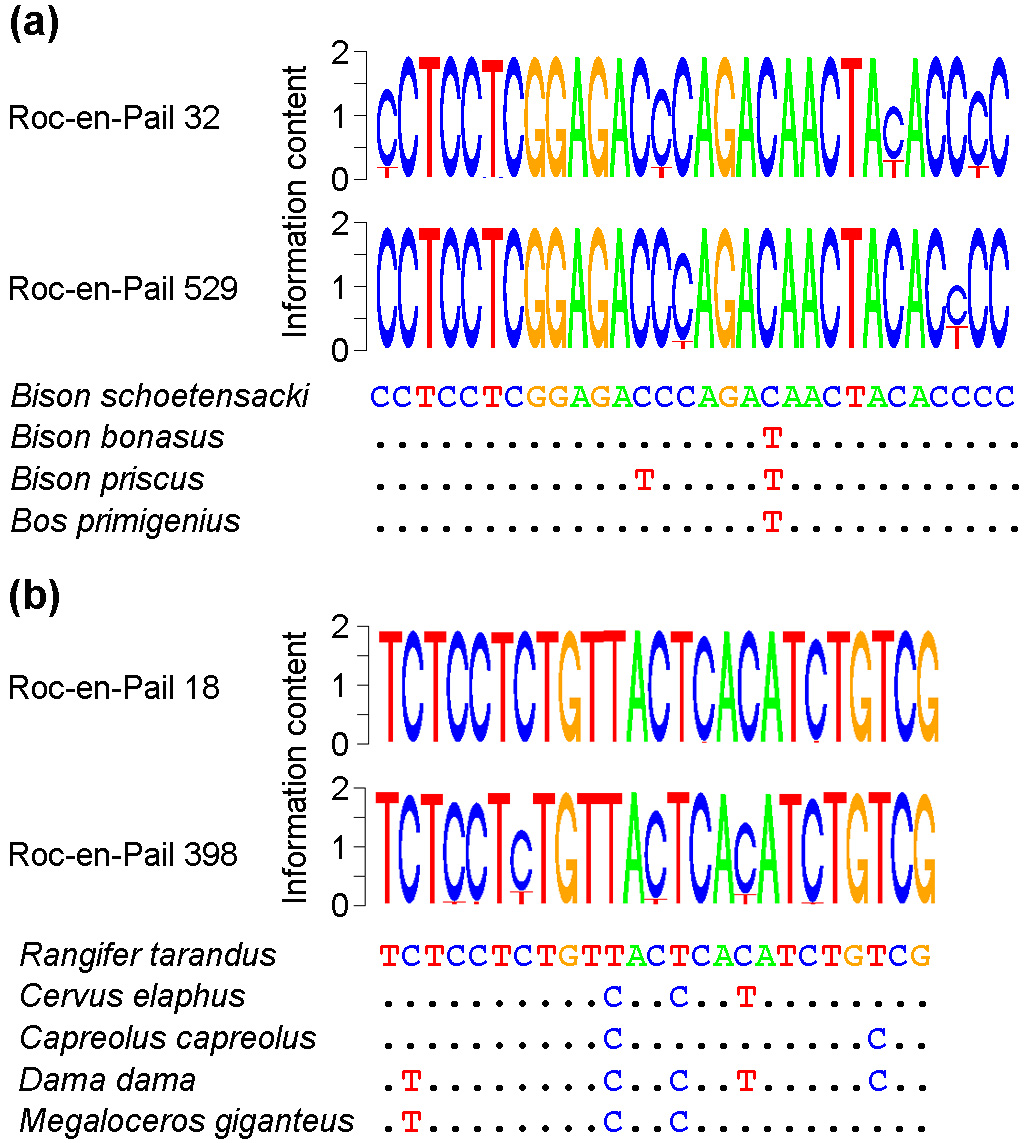

Supplement: S5 Fig — (a) Bovinae samples. The upper part of the figure displays sequence logos derived from 33,267 (Roc-en-Pail 32) and 30,091 (Roc-en-Pail 529) DNA reads. Only the sequence located between the PCR primers is shown. At each position, the upper letter corresponds to the predominant nucleotide. The lower part of the figure shows the orthologous reference sequences of the Bison schoetensacki (NC_033873), Bison pricus (NC_027233), Bison bonasus (NC_014044), and Bos primigenius (NC_013996) mitochondrial genomes. For both Roc-en-Pail samples, the consensus sequence is identical to the Bison schoetensacki reference sequence. Dots indicate sequence identity. (b) Cervidae sample. The upper part of the figure displays sequence logos derived from 23,481 (Roc-en-Pail 18) and 2,229 (Roc-en-Pail 398) DNA reads. The lower part of the figure shows the orthologous reference sequences of the Rangifer tarandus (NC_007703), Cervus elaphus (NC_007704), Capreolus capreolus (NC_020684), Dama dama (NC_020700) mitochondrial genomes, and the orthologous sequence of the Megaloceros giganteus (AM182645) mitochondrial cytB gene. For both Roc-en-Pail samples, the consensus sequence is identical to the Rangifer tarandus reference sequence. (JPG) [file pone.0230496.s005.jpg]

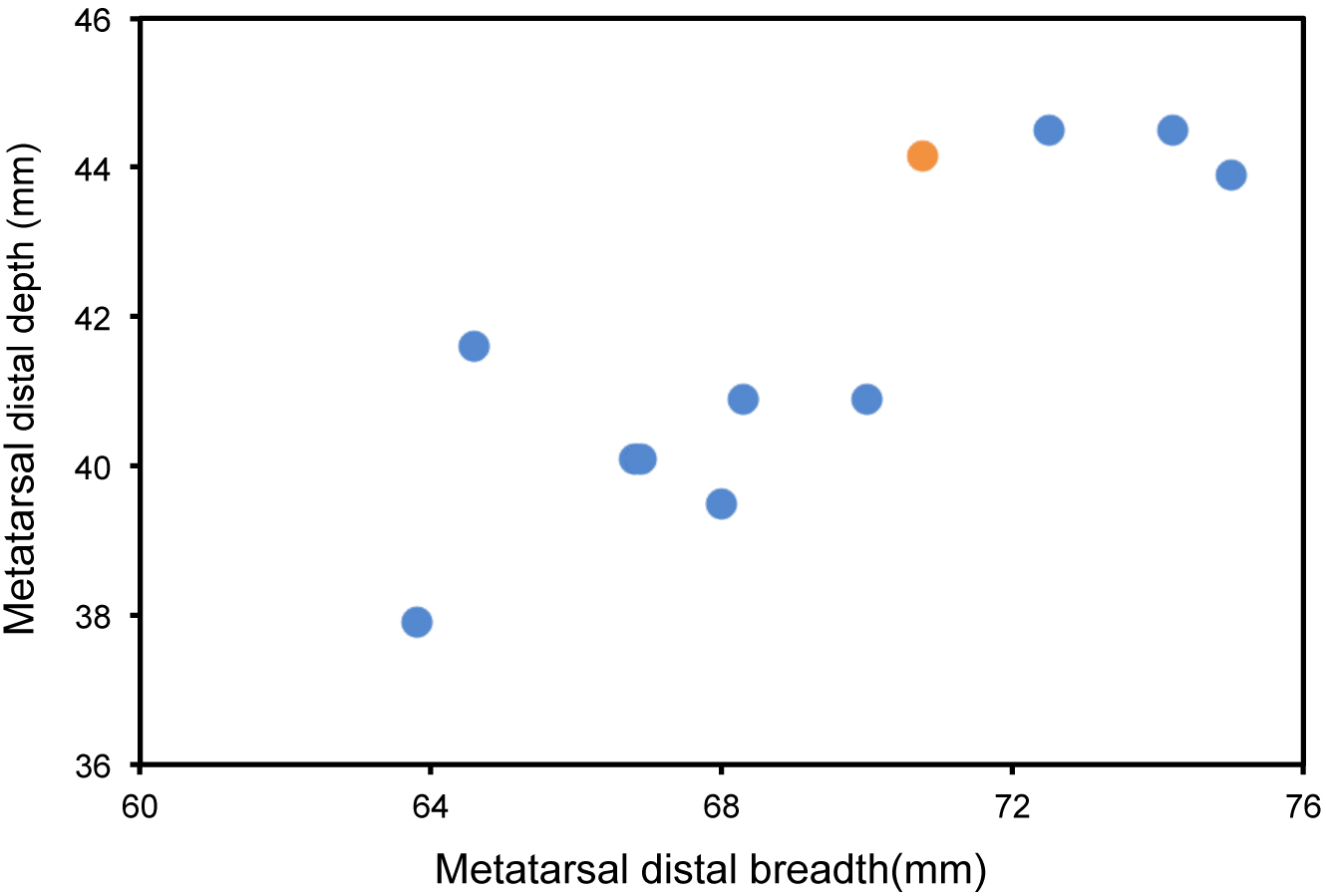

Supplement: S6 Fig — Comparative sizes of metatarsal distal ends of Roc-en-Pail 529 (orange dot) and Siréjol (blue dots) bison specimens. (JPG) [file pone.0230496.s006.jpg]

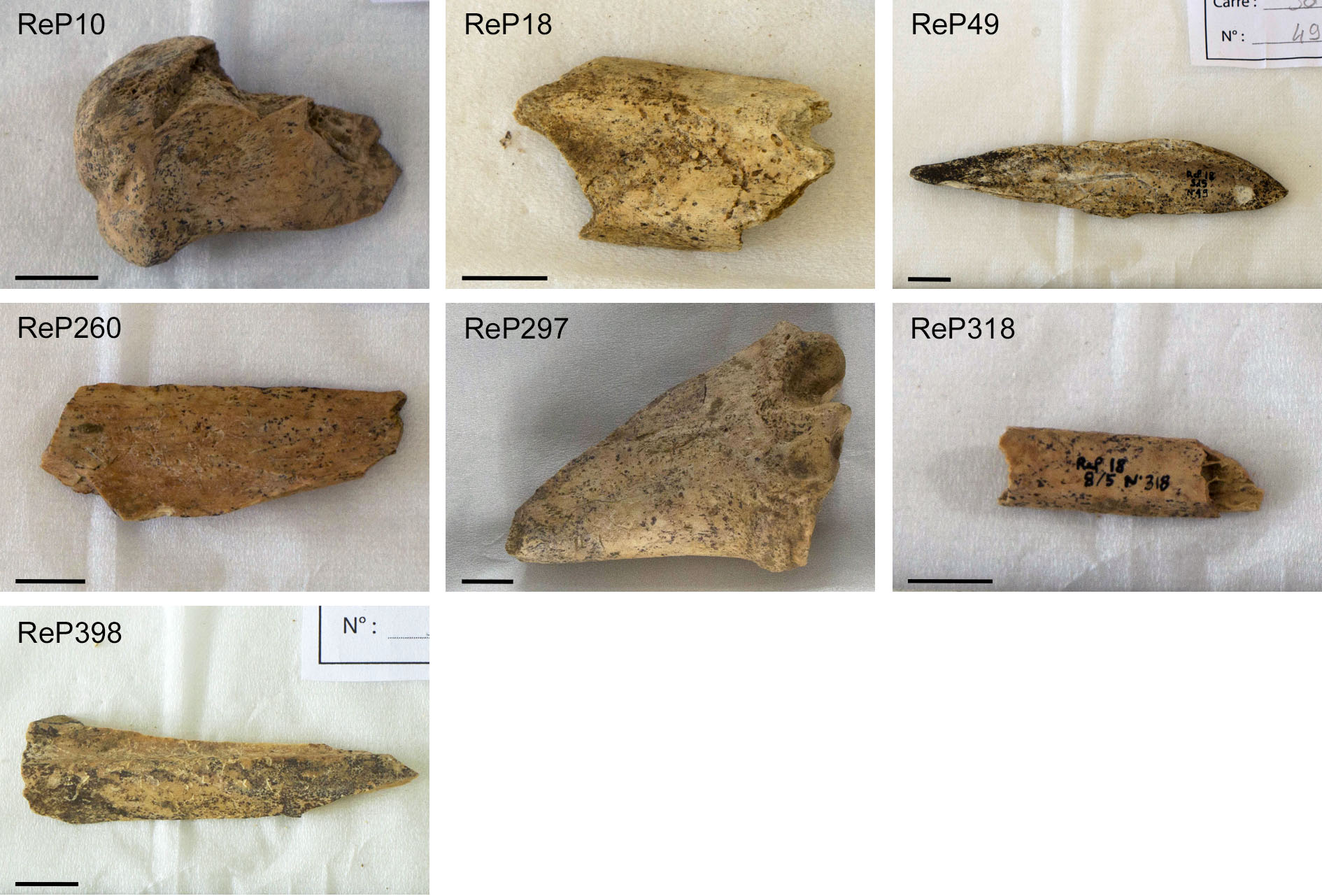

Supplement: S7 Fig — Scale bars: 1 cm. (JPG) [file pone.0230496.s007.jpg]
