# Supplementary material for: A mobile laboratory for ancient DNA analysis
Source: PLoS One. 2020 Mar 18;15(3):e0230496. doi: 10.1371/journal.pone.0230496 (PMC7080343; doi:10.1371/journal.pone.0230496)
Supplement: S1 Table — (DOCX) [file pone.0230496.s008.docx]

**Table S1.** Summary of samples analyzed and TaqMan assays performed in this study.

| Sample information | | |  | TaqMan assay | | | | | |
| --- | --- | --- | --- | --- | --- | --- | --- | --- | --- |
| Sample  name | Sample  type | Paleontology |  | *Bison priscus* | *Bison schoetensacki* | *Bos primigenius* | *Crocuta crocuta* | *Rangifer tarandus* | *Cervus elaphus* |
| Enlène 6170 | Bone | *Bison*/*Bos* |  | **-** | **-** | **-** | NT | NT | NT |
| Enlène 6171 | Bone | *Bison*/*Bos* |  | **-** | **-** | **-** | NT | NT | NT |
| Enlène 6172 | Bone | *Bison*/*Bos* |  | **+** | NT | NT | NT | NT | NT |
| Enlène 6173 | Bone | *Bison*/*Bos* |  | **+** | NT | NT | NT | NT | NT |
| Enlène 6174 | Bone | *Bison*/*Bos* |  | **-** | **-** | **-** | NT | NT | NT |
| Enlène 6175 | Bone | *Bison*/*Bos* |  | **-** | **-** | **-** | NT | NT | NT |
| Enlène 6176 | Bone | *Bison*/*Bos* |  | **+** | **-** | NT | NT | NT | NT |
| Enlène 6177 | Bone | *Bison*/*Bos* |  | **+** | **-** | NT | NT | NT | NT |
| Enlène 6178 | Bone | *Bison*/*Bos* |  | **+** | **-** | NT | NT | NT | NT |
| Enlène 6179 | Bone | *Bison*/*Bos* |  | **+** | NT | **-** | NT | NT | NT |
| Enlène 6180 | Bone | *Bison*/*Bos* |  | **-** | **-** | **-** | NT | NT | NT |
| Le Portel T1 | Coprolite | *Crocuta crocuta* |  | NT | **-** | NT | **-** | NT | NT |
| Le Portel T2 | Coprolite | *Crocuta crocuta* |  | **-** | **+** | **-** | **+** | **-** | **-** |
| Le Portel T3 | Coprolite | *Crocuta crocuta* |  | NT | **-** | NT | **-** | NT | NT |
| Le PortelT4 | Coprolite | *Crocuta crocuta* |  | NT | **-** | NT | **-** | NT | NT |
| Le Portel T5 | Coprolite | *Crocuta crocuta* |  | **-** | **-** | **-** | **+** | **-** | **-** |
| Le PortelT6 | Coprolite | *Crocuta crocuta* |  | **-** | **-** | **-** | **+** | **-** | **-** |
| Le Portel T7 | Coprolite | *Crocuta crocuta* |  | **-** | **-** | **-** | **+** | **-** | **-** |
| Le Portel T8 | Coprolite | *Crocuta crocuta* |  | **-** | **-** | **-** | **+** | **-** | **-** |
| Le Portel T9 | Coprolite | *Crocuta crocuta* |  | NT | **-** | NT | **-** | NT | NT |
| Le Portel T10 | Coprolite | *Crocuta crocuta* |  | NT | **-** | NT | **-** | NT | NT |
| Le Portel T11 | Coprolite | *Crocuta crocuta* |  | **-** | **-** | **-** | **-** | **-** | **-** |
| Le Portel T12 | Coprolite | *Crocuta crocuta* |  | **-** | **-** | **-** | **+** | **-** | **-** |
| Le Portel T13 | Coprolite | *Crocuta crocuta* |  | **-** | **-** | **-** | **+** | **-** | **-** |
| Le Portel T14 | Coprolite | *Crocuta crocuta* |  | **-** | **+** | **-** | **+** | **-** | **-** |
| Roc-en-Pail 4 | Bone | *Bison*/*Bos* |  | **-** | **-** | **-** | NT | NT | NT |
| Roc-en-Pail 12 | Bone | *Bison*/*Bos* |  | **-** | **-** | **-** | NT | NT | NT |
| Roc-en-Pail 32 | Bone | *Bison*/*Bos* |  | **-** | **+** | **-** | NT | NT | NT |
| Roc-en-Pail 33 | Bone | *Bovinae/Cervidae* |  | **-** | **-** | **-** | NT | **-** | **-** |
| Roc-en-Pail 37 | Bone | *Bovinae/Cervidae* |  | **-** | **-** | **-** | NT | **-** | **-** |
| Roc-en-Pail 44 | Bone | *Bison*/*Bos* |  | **-** | **-** | **-** | NT | NT | NT |
| Roc-en-Pail 54 | Bone | *Bovinae/Cervidae* |  | **-** | **-** | **-** | NT | **-** | **-** |
| Roc-en-Pail 118 | Bone | *Bovinae/Cervidae* |  | **-** | **-** | **-** | NT | **-** | **-** |
| Roc-en-Pail 181 | Bone | *Bison*/*Bos* |  | **-** | **-** | **-** | NT | NT | NT |
| Roc-en-Pail 182 | Tooth | *Bison*/*Bos* |  | **-** | **+** | **-** | NT | NT | NT |
| Roc-en-Pail 206 | Bone | *Bovinae/Cervidae* |  | **-** | **-** | **-** | NT | **-** | **-** |
| Roc-en-Pail 337 | Bone | *Bison*/*Bos* |  | **-** | **+** | **-** | NT | NT | NT |
| Roc-en-Pail 410 | Bone | *Bison*/*Bos* |  | **-** | **-** | **-** | NT | NT | NT |
| Roc-en-Pail 464 | Bone | *Bison*/*Bos* |  | **-** | **-** | **-** | NT | NT | NT |
| Roc-en-Pail 528 | Bone | *Bison*/*Bos* |  | **-** | **-** | **-** | NT | NT | NT |
| Roc-en-Pail 529 | Bone | *Bison*/*Bos* |  | **-** | **+** | **-** | NT | NT | NT |
| Roc-en-Pail 10 | Bone | *Rangifer tarandus* |  | NT | NT | NT | NT | **+** | **-** |
| Roc-en-Pail 11 | Bone | *Rangifer tarandus* |  | NT | NT | NT | NT | **-** | **-** |
| Roc-en-Pail 18 | Bone | *Rangifer tarandus* |  | NT | NT | NT | NT | **+** | **-** |
| Roc-en-Pail 29 | Bone | *Rangifer tarandus* |  | NT | NT | NT | NT | **-** | **-** |
| Roc-en-Pail 49 | Bone | *Rangifer tarandus* |  | NT | NT | NT | NT | **+** | **-** |
| Roc-en-Pail 75 | Bone | *Rangifer tarandus* |  | NT | NT | NT | NT | **-** | **-** |
| Roc-en-Pail 196 | Bone | *Rangifer tarandus* |  | NT | NT | NT | NT | **-** | **-** |
| Roc-en-Pail 231 | Bone | *Rangifer tarandus* |  | NT | NT | NT | NT | **-** | **-** |
| Roc-en-Pail 260 | Bone | *Rangifer tarandus* |  | NT | NT | NT | NT | **+** | **-** |
| Roc-en-Pail 297 | Bone | *Rangifer tarandus* |  | NT | NT | NT | NT | **+** | **-** |
| Roc-en-Pail 318 | Bone | *Rangifer tarandus* |  | NT | NT | NT | NT | **+** | **-** |
| Roc-en-Pail 321 | Bone | *Cervidae/Bovinae* |  | **-** | **-** | **-** | NT | **-** | **-** |
| Roc-en-Pail 398 | Bone | *Rangifer tarandus* |  | NT | NT | NT | NT | **+** | **-** |
| Roc-en-Pail 409 | Bone | *Rangifer tarandus* |  | NT | NT | NT | NT | **-** | **-** |

TaqMan assay: +, positive result; -, negative result; NT : not tested.
